# Exploring clinical indicator variations in stroke patients with multiple risk factors: focus on hypertension and inflammatory reactions

**DOI:** 10.1186/s40001-024-01653-6

**Published:** 2024-01-29

**Authors:** Jiejie Guo, Mei Tian, Yongang Li, Yitong Guo, Ting Zhang, Xuan Liu, Jinze Shen, Lin Zhang, Yueqi Yu, Ling Cao, Haiyan Gu, Yanfang Li, Shiwei Duan, Qinwen Wang

**Affiliations:** 1 Zhejiang Key Laboratory of Pathophysiology, NBU Health Science Center, Ningbo University, Ningbo, 315211 Zhejiang China; 2 Department of Clinical Laboratory, The Affiliated Wenling Hospital, Wenzhou Medical University, Wenling, 317500 Zhejiang China; 3 Department of Neurology, The Affiliated Wenling Hospital, Wenzhou Medical University, Wenling, 317500 Zhejiang China; 4 College of Pharmacy, Zhejiang University of Technology, Hangzhou, 310014 Zhejiang China; 5 Key Laboratory of Novel Targets and Drug Study for Neural Repair of Zhejiang Province, School of Medicine, Hangzhou City University, Hangzhou, 310015 Zhejiang China; 6 Ningbo Rehabilitation Hospital, Ningbo, 315040 China

**Keywords:** Stroke, Risk factors, Ischemic stroke subtype, Clinical indicators, Inflammatory reactions

## Abstract

**Background:**

Stroke stands as the second leading cause of death worldwide. Currently, extensive research has been conducted on stroke risk factors. However, when stroke patients contend with multiple risk factors, the impact on clinical indicators remains uncertain.

**Objectives:**

This study seeks to investigate potential significant variations among distinct ranges of clinical indicators in instances where stroke patients experience multiple risk factors and various ischemic stroke subtypes.

**Material and methods:**

The research encompassed 440 stroke patients admitted to the First People's Hospital of Wenling City, Zhejiang Province, China. These patients were classified based on the type and quantity of risk factors and subtypes of ischemic stroke they presented. The χ^2^ test was employed to assess the relationship between the risk of comorbid diseases and clinical indicators in stroke patients.

**Results:**

The results of our study have underscored a significant correlation between various comorbid risk factors in stroke patients and the patients' age (*P* < 0.010). Furthermore, we observed noteworthy disparities in the plasma levels of IL-2, IL-4, IL-6, IL-10, TNF-α, and INF-γ between patients devoid of risk factors and those presenting with comorbid risk factors associated with stroke. Significant differences in INF-γ were observed between the two subtypes of ischemic stroke, namely lacunar infarction and cardioembolic stroke.

**Conclusion:**

Age is correlated with an elevated risk of stroke. Individuals exhibiting multiple stroke risk factors and diverse ischemic stroke subtypes commonly present with abnormal lipid levels and imbalances in Th1/Th2 cytokines. These factors significantly contribute to the onset and progression of stroke. Furthermore, inflammatory responses, particularly those induced by atherosclerosis, play a pivotal role in the genesis of stroke and exert a substantial influence on its prognosis.

**Supplementary Information:**

The online version contains supplementary material available at 10.1186/s40001-024-01653-6.

## Introduction

Stroke, a formidable global health challenge, ranks as the second leading cause of death worldwide, accounting for a staggering fatalities [1]. It manifests as either a cerebral hemorrhage or ischemic event, inflicting damage upon cerebral blood vessels and precipitating localized or widespread brain tissue impairment, as evidenced by research [2, 3]. Stroke is classified into two main types based on the nature of blood vessel damage: ischemic stroke and hemorrhagic stroke [2]. Ischemic stroke, constituting 71% of all strokes globally occurs when blood flow to the brain is obstructed [2, 4]. Stroke is a multifaceted affliction, influenced by a myriad of factors encompassing those beyond one's control, such as age, gender, genetics, and birth weight, as well as modifiable factors, including hypertension, diabetes, dyslipidemia, atrial fibrillation, smoking, excessive alcohol consumption, obesity, and physical inactivity [1] Timely recognition and diligent management of these risk factors constitute pivotal strategies in the prevention and treatment of stroke.

Notably, research findings underscore that when individuals grappling with stroke concurrently exhibit two or more risk factors, their susceptibility to adverse outcomes is exponentially heightened [5]. Consequently, conducting a comprehensive assessment and proficiently managing clinical indicators among stroke patients harboring one or more risk factors significantly contributes to their therapeutic journey and overall prognosis. Among the diverse spectrum of stroke risk factors, hypertension reigns supreme as the foremost contributor [6]. Hypertension substantially amplifies the risk of atherosclerosis, culminating in the formation of atherosclerotic plaques that can precipitate intra-arterial embolisms or acute cerebrovascular occlusions, thereby instigating stroke events [7].

Diabetes Mellitus represents a chronic metabolic disorder characterized by hyperglycemia, with studies unequivocally revealing a heightened ischemic stroke risk among diabetic patients compared to their non-diabetic counterparts [8, 9]. Furthermore, acute ischemic strokes may precipitate abrupt spikes in blood sugar levels, impinging upon treatment efficacy and prognostic outcomes [10].

Atrial fibrillation a prevalent cardiac arrhythmia, significantly elevates the risk of ischemic stroke [11]. In contrast to stroke patients without atrial fibrillation, those with this arrhythmia face a twofold greater risk of disability and mortality, with atrial fibrillation intricately intertwined with unfavorable prognostic trajectories in stroke patients [12].

Hyperlipidemia can perturb vascular endothelial function and disrupt the equilibrium of pro-fibrinolytic and antithrombotic factors, thereby fostering structural vessel wall damage and functional aberrations, thus instigating the atherosclerotic cascade leading to stroke onset [13, 14].

Hyperhomocysteinemia emerges as a closely correlated factor in stroke [15–18]. Notably, hyperhomocysteinemia is significantly intertwined with deficiencies in essential vitamins [19]. Insufficient vitamin B12 levels precipitate perturbations in immune homeostasis, potentially paving the way for atherosclerotic diseases that can culminate in strokes [20]. The dearth of vitamin B12 can also intricately shape stroke pathogenesis, its severity, and ultimate prognostic outcomes [21].

Ischemic stroke typically arises from a blood clot or embolism, leading to a disruption in the blood supply to a specific area of the brain [22]. Blood biomarkers play a crucial role in the etiology of diverse ischemic stroke subtypes [23]. They can instigate inflammatory responses, thrombosis, and antifibrinolysis, exacerbate atherosclerosis, and cause damage to endothelial cells [24]. Consequently, these biomarkers contribute to a more severe neurological outcome following an ischemic stroke event [22].

While numerous studies have underscored the pivotal role of various clinical indicators in distinct ischemic stroke subtypes and highlighted the substantial impact of the coexistence of one or more risk factors on the treatment and prognosis of stroke patients, there is a paucity of research on potential differences among the ranges of clinical indicators in patients with stroke who concurrently harbor one or more risk factors and suffer from varying ischemic stroke subtypes. This study seeks to investigate whether stroke patients, burdened with multiple risk factors, exhibits significant differences in clinical indicators across diverse ischemic stroke subtypes. The objective is to furnish insights that could pave the way for effective personalized treatments tailored to the needs of stroke patients grappling with multiple risk factors.

## Materials and methods

### Study population

A comprehensive cohort of 440 stroke patients was meticulously gathered from the First People’s Hospital of Wenling City, Zhejiang Province, China, spanning the period from February 2021 to February 2023. These individuals exhibited a broad age spectrum, spanning from 15 to 93 years, with a mean age of 68 years. Notably, one sample within the dataset lacked critical clinical information, while gender data for two samples were also incomplete. The gender distribution within the study cohort comprised 302 males and 135 females. It is paramount to underscore that this study was conducted under the auspices of approval from the Ethics Review Committee of Wenling First People’s Hospital (Approval Number: KY-2019-2091-01), and prior to inclusion, all subjects, and their respective families, provided unequivocal informed consent.

### Diagnostic criteria and definitions

Screening for risk factors was conducted based on well-defined diagnostic criteria. Hypertension: Diagnosed when diastolic blood pressure (DBP) registered ≥ 90 mm Hg, systolic blood pressure (SBP) exceeded 140 mm Hg, as evidenced by three separate measurements, and/or when patients were actively receiving antihypertensive medications within a 2 week timeframe. Diabetes: Confirmed in cases of fasting blood glucose (FBG) levels ≥ 7.0 mmol/L, 2 h postprandial blood glucose (2 h-FBG) levels ≥ 11.1 mmol/L, or postprandial hemoglobin (HbA1c) levels reaching or surpassing 6.5%. Atrial Fibrillation: Ascertained in individuals exhibiting atrial fibrillation or manifesting obvious irregular heartbeat patterns [25]. Hyperlipidemia: Defined by an amalgamation of metrics, including total cholesterol (TC) levels ≥ 5.72 mmol/L, triglyceride (TG) levels ≥ 1.70 mmol/L, high-density low lipoprotein cholesterol (HDL-C) levels < 1.0 mmol/L, and low-density lipoprotein cholesterol (LDL-C) levels ≥ 3.4 mmol/L. Hyperhomocysteinemia: Noted when Hyperhomocysteinemia levels measured ≥ 15 μmol/L. It is pertinent to recognize the close linkage between Hyperhomocysteinemia and a spectrum of conditions [15–18]. These stringent diagnostic criteria and definitions served as the foundation upon which risk factors were identified and analyzed within the study cohort, furnishing a robust framework for investigation and assessment.

### Inclusion criteria

**Fig. 1:**
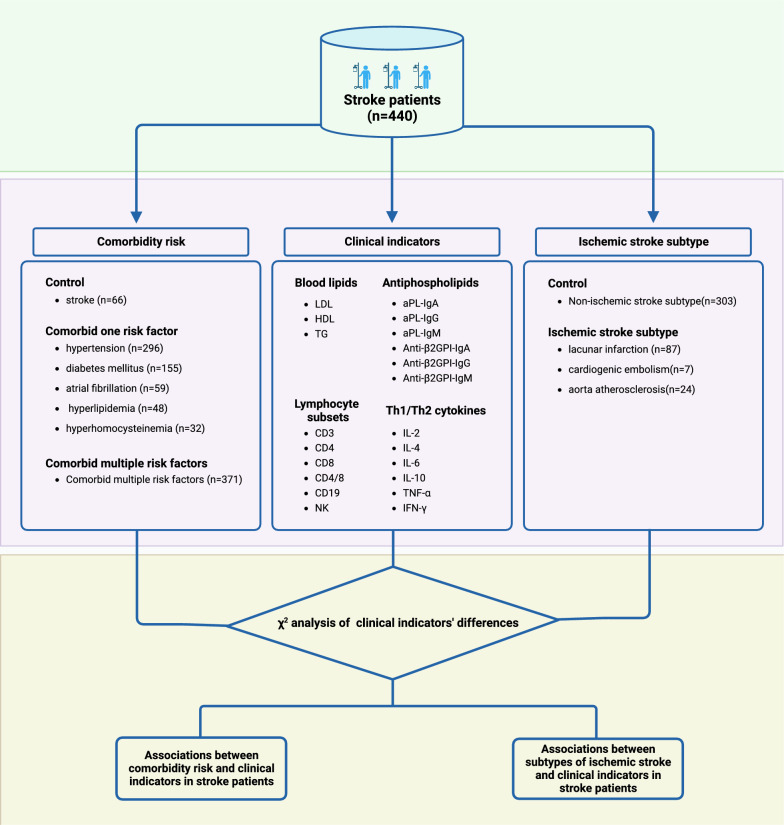
Flowchart of correlation between stroke patients and clinical indicators. Our study classified stroke patients into single and multiple risk factor groups, considering hypertension, diabetes, hyperlipidemia, atrial fibrillation, and hyperhomocysteinemia. Co-morbidity denotes stroke presence with multiple risk factors. Additionally, we conducted screenings on patients presenting with specific subtypes of ischemic stroke within the larger cohort of stroke patients. Our study delineates three primary subtypes of ischemic stroke: lacunar infarction, cardioembolic, and large artery atherosclerosis. Clinical indicators were grouped into lymphatic subcellular, Th1/Th2 lymphocyte factor, blood lipid, and anticardiolipin antibody tests. We used the *χ*^2^ test to assess the correlation between comorbid disease risk and clinical indicators in stroke patients

The criteria for participant inclusion in this study adhere to the standards established in 2018 [26]. The collected demographic information encompasses key aspects such as age, gender, and education level. The focus of investigation extends to risk factors associated with stroke, including hypertension, diabetes, atrial fibrillation, hyperlipidemia, and hyperhomocysteinemia. The TOAST (Trial of Org 10172 in Acute Stroke Treatment) study [27] focuses on the ischemic stroke subtype classification, delineating categories such as large artery atherosclerosis, lacunar infarction, cardioembolic, unexplained infarction, and infarction of unusual etiology [28] (As show in Table 1 and Fig. 1).

**Table 1:** Clinical indicator profiles in stroke patients with comorbidities

Variable	Stroke (*n* = 66)	Combid risk factor					*P*	Co-morbidity of multiple risk factors	P*	Stroke subtypes				*P#*
		Hypertension (*n* = 296)	Diabetes mellitus (*n* = 155)	Hyperlipidemia (*n* = 59)	Atrial fibrillation (*n* = 48)	Hyperhomocysteinaemia (*n* = 32)		Multiple risk factors (*n* = 371)		Non-ischemic stroke subtype (*n* = 303)	Lacunar blockage (*n* = 87)	Cardiogenic embolism (*n* = 7)	Aorta atherosclerosis (*n* = 24)	
Age, median (range) (years)	66 (15–89)	68 (33–93)	68 (35–93)	66 (46–90)	75 (52–90)	64 (34–87)	< 0.001*	68 (33–93)	0.010*	68 (15–93)	65 (35–91)	81 (72–90)	65 (36–85)	0.130
Female	22	96	49	11	18	3	0.065	112	0.377	98	24	1	7	0.242
Blood lipid profile														
LDL (mg/dl)	3.184 ± 15.441	2.861 ± 0.816	2.899 ± 0.936	3.614 ± 0.971	2.379 ± 0.994	2.862 ± 0.831	< 0.001*	2.869 ± 0.889	0.033*	2.930 ± 0.906	2.903 ± 0.815	4.730 ± 2.160	2.764 ± 0.756	< 0.001*
HDL (mg/dl)	1.247 ± 0.506	1.104 ± 0.258	1.049 ± 0.234	1.159 ± 0.215	1.024 ± 0.24	1.141 ± 0.244	< 0.001*	1.104 ± 0.265	0.039*	1.137 ± 0.332	1.101 ± 0.233	1.004 ± 0.211	1.042 ± 0.183	< 0.001*
TG (mg/dl)	1.315 ± 0.606	1.634 ± 1.603	1.936 ± 2.018	2.61 ± 2.716	1.156 ± 0.865	1.645 ± 1.018	< 0.001*	1.574 ± 1.477	0.057	1.504 ± 1.447	1.605 ± 1.207	1.341 ± 1.071	1.642 ± 1.220	< 0.001*
Antiphospholipid antibody														
aPL-IgA (IU/mL)	6.45 ± 6.627	7.7 ± 11.38	23.59 ± 41.481	3.667 ± 0.502	0	0	0.112	15.953 ± 35.767	0.255	13.232 ± 32.988	14.700 ± 22.273	/	/	0.183
aPL-IgG (IU/mL)	2.007 ± 0.759	2.299 ± 3.422	2.547 ± 3.538	2.22 ± 2.546	3.471 ± 5.864	1.852 ± 0.514	< 0.001*	2.418 ± 3.616	0.06	2.062 ± 2.143	3.146 ± 5.383	2.133 ± 0.808	2.675 ± 4.300	0.002*
aPL-IgM (IU/mL)	6.636 ± 5.514	6.448 ± 7.384	6.417 ± 8.078	8.462 ± 10.642	4.874 ± 3.638	6.908 ± 4.898	< 0.001*	6.625 ± 7.493	< 0.001*	7.106 ± 8.326	5.678 ± 4.22	8.700 ± 4.950	4.625 ± 3.641	0.005*
Anti-β2GPI-IgA (IU/mL)	3.18 ± 2.12	3.996 ± 8.002	3.317 ± 2.23	2.908 ± 1.627	2.100 ± 0.494	2.367 ± 0.804	< 0.001*	3.717 ± 7.371	0.05	3.957 ± 8.089	2.923 ± 1.672	/	2.700 ± 1.322	0.066
Anti-β2GPI-IgG (IU/mL)	4.563 ± 4.861	4.052 ± 5.161	4.777 ± 6.302	3.441 ± 1.775	2.656 ± 0.480	3.688 ± 1.697	< 0.001*	4.338 ± 6.081	0.016*	4.254 ± 5.223	5.554 ± 8.343	2.700 ± 0.566	3.050 ± 0.547	0.009*
Anti-β2GPI-IgM (IU/mL)	19.954 ± 31.410	28.239 ± 64.561	27.938 ± 6.302	40.103 ± 91.300	9.527 ± 9.770	18.045 ± 25.706	0.002*	27.664 ± 60.055	0.102	29.649 ± 65.543	20.435 ± 30.147	15.200 ± 14.142	19.514 ± 36.979	0.006*
Lymphocyte subpopulation														
CD3 (cells/mm^3^)	67.952 ± 12.599	70.675 ± 11.299	72.514 ± 10.337	73.103 ± 9.128	65.879 ± 13.848	73.398 ± 7.324	< 0.001*	70.587 ± 11.219	0.012*	69.666 ± 11.603	71.217 ± 10.038	66.300 ± 17.255	74.284 ± 8.825	< 0.001*
CD4 (cells/mm^3^)	42.845 ± 12.004	44.607 ± 11.299	45.434 ± 10.520	46.200 ± 9.171	40.131 ± 11.883	46.503 ± 8.195	< 0.001*	44.424 ± 10.953	0.012*	43.808 ± 11.544	45.190 ± 9.609	40.686 ± 13.312	47.372 ± 8.779	< 0.001*
CD8 (cells/mm^3^)	20.768 ± 9.545	21.971 ± 8.345	22.670 ± 8.528	22.780 ± 7.071	21.811 ± 9.680	22.768 ± 8.310	< 0.001*	22.084 ± 8.258	0.020*	21.647 ± 8.650	22.337 ± 7.123	23.829 ± 19.126	21.689 ± 7.008	< 0.001*
CD4/8 (cells/mm^3^)	2.631 ± 2.203	2.400 ± 1.226	2.344 ± 1.123	2.312 ± 1.010	2.267 ± 1.563	2.430 ± 1.250	< 0.001*	2.38 ± 1.254	0.032*	2.469 ± 1.544	2.288 ± 1.056	3.119 ± 3.096	2.381 ± 0.776	< 0.001*
CD19 (cells/mm^3^)	13.446 ± 7.535	12.640 ± 6.944	12.917 ± 6.439	11.549 ± 5.690	12.697 ± 8.409	11.018 ± 5.646	< 0.001*	12.628 ± 7.049	0.020*	13.009 ± 7.530	11.540 ± 5.470	9.771 ± 5.449	14.859 ± 6.932	0.002*
NK (cells/mm^3^)	18.750 ± 11.081	16.364 ± 9.790	14.481 ± 9.020	15.153 ± 8.258	21.027 ± 11.956	15.047 ± 7.826	< 0.001*	16.466 ± 9.778	0.041*	16.912 ± 9.894	17.102 ± 10.150	22.843 ± 15.432	11.578 ± 5.517	0.005*
Th1/Th2 Lymphocytes														
IL-2 (cells/mm^3^)	1.850 ± 0.741	1.889 ± 0.572	1.881 ± 0.544	1.877 ± 0.490	1.932 ± 0.604	1.915 ± 0.646	< 0.001*	1.894 ± 0.564	0.007*	1.900 ± 0.554	1.900 ± 0.713	1.800 ± 0.379	1.823 ± 0.595	< 0.001*
IL-4 (cells/mm^3^)	2.417 ± 0.747	2.439 ± 0.585	2.458 ± 0.558	2.535 ± 0.501	2.497 ± 0.616	2.389 ± 0.469	< 0.001*	2.442 ± 0.594	0.003*	2.471 ± 0.607	2.446 ± 0.775	2.300 ± 0.707	2.341 ± 0.482	< 0.001*
IL-6 (cells/mm^3^)	9.820 ± 33.635	17.872 ± 145.321	28.416 ± 194.541	18.024 ± 56.436	12.062 ± 20.966	5.811 ± 7.638	0.005*	16.884 ± 0.594	0.165	17.534 ± 143.329	7.688 ± 23.417	6.967 ± 4.278	20.831 ± 75.169	0.032*
IL-10 (cells/mm^3^)	3.344 ± 1.047	6.830 ± 51.746	9.644 ± 69.296	3.008 ± 0.521	3.394 ± 1.013	3.041 ± 1.304	0.007*	6.082 ± 45.901	0.18	6.898 ± 51.500	3.148 ± 0.966	3.217 ± 0.624	3.064 ± 0.564	0.023*
TNF-α (cells/mm^3^)	3.017 ± 1.578	2.778 ± 0.874	2.807 ± 0.906	2.937 ± 0.883	2.853 ± 1.049	2.519 ± 0.707	< 0.001*	2.784 ± 0.88	0.026*	2.855 ± 0.914	2.901 ± 1.506	2.717 ± 0.799	2.618 ± 0.673	< 0.001*
IFN-γ (cells/mm^3^)	2.766 ± 1.113	3.004 ± 2.536	3.235 ± 3.639	2.850 ± 0.653	3.979 ± 5.964	2.605 ± 0.654	< 0.001*	3.058 ± 2.544	0.032*	2.927 ± 1.599	2.836 ± 0.956	9.333 ± 13.770	2.742 ± 0.622	0.07

Values are presented as mean ± SD. “*” represent significant differences. *P*: A comparison between stroke patients without risk factors and stroke patients with a single risk factor. P*: A comparison between stroke patients without risk factors and stroke patients with multiple risk factors

### Lymphocyte subset detection

To evaluate lymphocyte subsets, flow cytometry was employed to detect distinct cell populations within the test sample, including total T cells (CD3), helper T cells (CD4), suppressor T cells (CD8), B cells (CD19), and NK (CD56) cells. To facilitate this, 5 μl of CD45/CD4/CD8/CD3 (FITC/RD1/ECD/PC5) antibody (Beckman Coulter, Inc., Brea, California, USA) and 5 μl of CD45/CD56/CD19/CD3 (FITC/RD1/ECD/PC5) antibodies were added to two separate flow sample tubes. Subsequently, 100 μl of EDTA-K2 anticoagulant blood (Gongdong Medical Co., Ltd., Taizhou, Zhejiang, China) was introduced to each tube, followed by thorough mixing and incubation in darkness for 20 min. Subsequently, 0.5 ml of hemolysin was added to each tube, thoroughly mixed, and incubated at room temperature (18–25 °C) in darkness for an additional 10 min. Afterward, centrifugation at 3000 rpm for 5 min was performed to discard the supernatant, and 500 μl of PBS solution (Beckman) was added, vortexed, and mixed. Finally, the machine was employed to carry out the detection process.

### Th1/Th2 lymphocyte cytokine detection

The capture microsphere mixture (A) sourced from the kit (Cell-genebio Co., Ltd., Hangzhou, Zhejiang, China) was meticulously prepared by vortexing and subsequently placing 25 μl into a sample tube, which was appropriately labeled. Post-centrifugation at 2000 rpm for 5 min, the supernatant was discarded, and the microspheres were re-suspended in an equal volume of liquid from microsphere buffer (H) (cell-genebio). Vortexing was employed to ensure uniformity, followed by incubation in darkness for 30 min. The standard tube (B) (cell-genebio) from the kit was then removed and transferred to the sample tube. Following this, 2 ml of sample diluent was introduced, left to stand for 15 min, and marked as the highest standard concentration. The preparation of diluted standards and test samples adhered to experimental requisites. Subsequently, an appropriate quantity of fluorescence detection reagent (C) (cell-genebio) was added to all standard and test sample tubes. Capture microspheres that had undergone incubation were extracted, thoroughly mixed, and introduced into all standard and test sample tubes. Vortexing ensured homogeneity, followed by incubation at room temperature in darkness for 2.5 h. Post-incubation, each tube was subjected to washing by adding an adequate amount of PBS solution, followed by centrifugation at 2000 rpm for 5 min, with subsequent supernatant discarding. A final step included adding 100 μl PBS solution to each tube, resuspending by shaking, and utilizing the machine for data acquisition. The acquired data were imported into FCAP (3.0.1) (Beckman) software for standard curve generation and measurement of test sample results.

### Measurement of blood lipid indicators

For the measurement of blood lipid indicators, venous blood was collected in the early morning after a fasting period of 8–10 h, followed by centrifugation at 4000 rpm for 10 min. The supernatant was then retrieved, and triglycerides (TG), low-density lipoprotein (LDL), and high-density lipoprotein (HDL) levels were determined using the Beckman Coulter AU5800 Automatic Biochemical Analyzer (Beckman).

### Antiphospholipid antibody indicator determination

The evaluation of antiphospholipid antibody indicators was executed utilizing a chemiluminescence instrument (iFlash 3000-C) (YHLO Co., Ltd., Longgang, Shenzhen, China) in conjunction with an antiphospholipid antibody detection kit (YHLO). This encompassed the determination of various parameters, including anticardiolipin antibody (aPL-IgA), anticardiolipin antibody (aPL-IgG), anticardiolipin antibody (aPL-IgM), anti-β2 glycoprotein I antibody (Anti-β2GPI-IgG), anti-β2 glycoprotein I antibody (Anti-β2GPI-IgG), and anti-β2 glycoprotein I antibody (Anti-β2GPI-IgG).

### Statistical methods

The initial phase of data analysis involved meticulous screening to eliminate any blank data entries. Only data points falling within the specified range of clinical indicators were retained for further analysis. Subsequently, the relevant data were organized and tabulated to facilitate subsequent statistical procedures. For non-normally distributed continuous data, the Mann–Whitney *U* test was applied. Conversely, normally distributed continuous variables were assessed using the Student’s *t*-test. The presentation of all data adhered to the convention of mean values along with their respective standard deviations or ranges. To investigate the relationship between clinical indicators of stroke patients as independent variables and the range of each clinical indicator as dependent variables, a *χ*^2^ test was conducted. The selection of the specific *χ*^2^ test variant depended on the sample size and expected cell counts. When the total sample size of the two groups (*n*) was ≥ 40, and the expected count (theoretical number, *T*) in all cells was ≥ 5, the Pearson *χ*^2^ test was employed. In cases where the obtained p-value was approximately 0.05, Fisher's exact test was utilized. When the sample size (*n*) was ≥ 40 but the expected count (*T*) fell within the range of 1–5, a continuity correction was applied. In instances where either the sample size (*n*) was less than 40 or the expected count (*T*) was less than 1, Fisher’s exact test was employed. For scenarios involving multiple rows and multiple lists, Fisher’s exact test was directly applied. The analysis sought to determine the significance of disparities between clinical indicators within the normal range and those within the abnormal range, distinguishing between comorbid and non-comorbid diseases. A threshold of *P* < 0.05 was deemed indicative of a statistically significant difference. The entire process of data analysis and statistical computation was performed using SPSS 26.0 (IBM, Chicago, Illinois, USA) software, ensuring rigor and precision in the analytical procedures.

## Results

### Baseline determination

Table 1 provides an overview of the baseline characteristics of stroke patients. It is evident that stroke predominantly afflicts individuals in the middle-aged and elderly demographic, particularly those aged 60 and above. Notably, the data indicate a higher incidence of stroke in women as compared to men. Additionally, there exist substantial disparities among nearly all clinical indicators for various comorbid diseases. Interestingly, hypertension stands out as the predominant comorbidity, demonstrating a significantly higher prevalence than other related conditions.

### Correlation between comorbid risk factors and clinical indicators in stroke patients

In our study, a comprehensive analysis was conducted on clinical data from a total of 439 cases, with the exclusion of one case due to missing data. The stroke patients were categorized based on the presence of various comorbid diseases, as summarized in Table 1. Specifically, the cohort comprised 66 stroke patients without any risk factors, 296 stroke patients with hypertension, 155 stroke patients with diabetes, and 59 stroke patients with hyperlipidemia. Additionally, there were 48 stroke patients with atrial fibrillation and 32 with hyperhomocysteinemia. To evaluate the association between different comorbidities and stroke, we performed a *χ*^2^ test on the prevalence of each comorbidity. The objective was to investigate the correlation between various clinical indicators at different concentration levels among stroke patients with and without comorbid diseases.

**Fig. 2:**
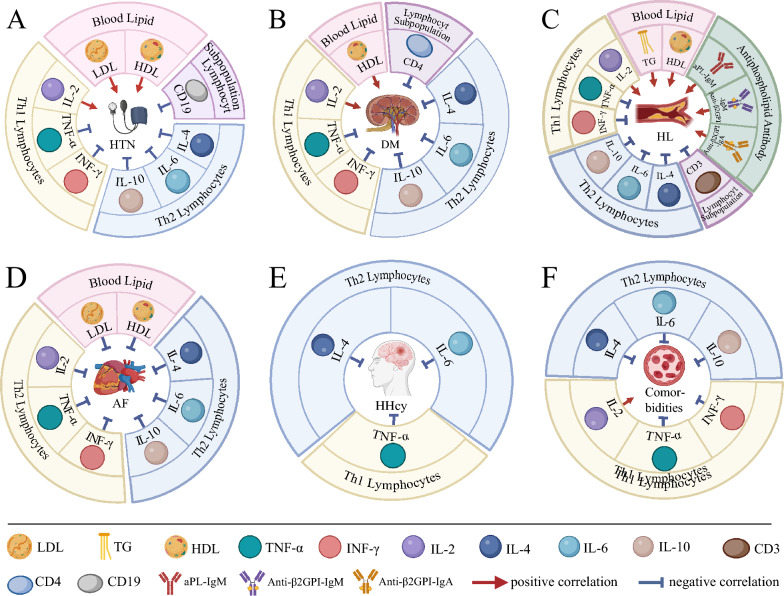
Correlation analysis of clinical indicators with comorbid risk factors in stroke patients. **A** Comorbid Hypertension. Positively correlated: LDL, HDL, IL-2; Negatively correlated: CD19, IL-4, IL-6, IL-10, TNF-α, INF-γ. **B** Comorbid Diabetes. Positively related: HDL, IL-2; Negatively correlated: CD4, IL-4, IL-6, IL-10, TNF-α, INF-γ. **C** Comorbid Hyperlipidemia. Positively related: HDL, IL-2, TG, aPL-IgM, Anti-β2GPI-IgA, Anti-β2GPI-IgM; Negatively correlated: HDL, CD3, IL-4, IL-6, IL-10, TNF-α, INF-γ. **D** Comorbid Atrial Fibrillation. Negatively correlated: LDL, HDL, IL-4, IL-6, IL-10, TNF-α, INF-γ. **E** Comorbid Hyperhomocysteinemia. Negatively related: IL-4, IL-6, TNF-α. **F** Multiple Comorbid Risk Factors: Positively associated: IL-2; Negatively correlated: IL-4, IL-6, IL-10, TNF-α, INF-γ

We analyzed the age and 20 clinical indicators of stroke patients with comorbid hypertension, and the results are presented in Additional file 1: Table S2. Among these indicators, a total of 9 showed significant differences. Table 2 showcases the significant variations in plasma levels of LDL, HDL, IL-2, CD19, IL-4, IL-6, IL-10, TNF-α, and INF-γ between stroke patients with and without comorbid hypertension. Specifically, elevated levels of LDL, HDL, and IL-2 were positively linked to the presence of comorbid hypertension in stroke patients (LDL: *P* = 0.040, *χ*^2^ = 5.068; HDL: *P* = 0.006, *χ*^2^ = 7.658; IL-2: *P* < 0.001, *χ*^2^ = 21.879). Conversely, decreased concentrations of CD19, IL-4, IL-6, IL-10, TNF-α, and INF-γ were inversely correlated with the occurrence of hypertension comorbid with stroke (CD19: *P* = 0.039, *χ*^2^ = 4.277; IL-4: *P* < 0.001, *χ*^2^ = 39.200; IL-6: *P* < 0.001, *χ*^2^ = 40.422; IL-10: *P* < 0.001, *χ*^2^ = 30.744; TNF-α: *P* < 0.001, *χ*^2^ = 41.178; INF-γ: *P* < 0.001, *χ*^2^ = 40.468) (Fig. 2A).

**Table 2:** Relationships between hypertension comorbidity and clinical indicators in stroke patients

Variable	Comorbidity		*χ*^2^	*P*	*χ*^2^	P*
	No	Yes				
LDL						
1.89–4.21 mg*/dl*	52	237			5.428	0.066
> 4.21* mg/dl*	8	13	5.068	0.040*		
< 1.89* mg/dl*	6	32	0.112	0.825		
HDL						
1.03–1.55* mg/dl*	32	162			7.622	0.022*
> 1.55* mg/dl*	9	13	7.658	0.010*		
< 1.03* mg/dl*	25	93	1.082	0.365		
CD19						
6.4–22.4%	44	226			5.34	0.069
> 22.4%	7	19	1.878	0.272		
< 6.4%	15	38	4.277	0.039*		
IL-2						
1.1–9.8 pg/ml	54	214			21.985	< 0.001*
> 9.8 pg/ml	6	0	21.879	< 0.001*		
< 1.1 pg/ml	6	23	0.005	1		
IL-4						
0.1–3 pg/ml	50	193			40.915	< 0.001*
> 3 pg/ml	4	21	0.295	0.794		
< 0.1 pg/ml	12	0	39.2	< 0.001*		
IL-6						
1.7–16.6 pg/ml	52	206			40.651	< 0.001*
> 16.6 pg/ml	2	8	0	1		
< 1.7 pg/ml	12	0	40.422	< 0.001*		
IL-10						
2.6–4.9 pg/ml	52	200			40.58	< 0.001*
> 4.9 pg/ml	2	12	0.33	0.742		
< 2.6 pg/ml	12	2	30.744	< 0.001*		
TNF-α						
0.1–5.2 pg/ml	52	210			41.243	< 0.001*
> 5.2 pg/ml	2	4	0.663	0.348		
< 0.1 pg/ml	12	0	41.178	< 0.001*		
INF-γ						
1.6–17.3 pg/ml	54	213			40.877	< 0.001*
> 17.3 pg/ml	0	1	0.253	1		
< 1.6 pg/ml	12	0	40.468	< 0.001*		

More data can be found in Additional file 1: Table S2. *P*: A comparison of different ranges of clinical indicators, and a comparison of clinical indicators that are above or below the normal range with clinical indicators that are within the normal range. P*: Multiple list comparisons between different ranges of clinical indicators

We investigated the ages of stroke patients with comorbid diabetes along with 20 clinical indicators, and the results are detailed in Additional file 1: Table S3. Among these indicators, a total of 9 exhibited significant differences. Table 3 presents data concerning the age of the patients and plasma levels of HDL, IL-2, CD4, IL-4, IL-6, IL-10, TNF-α, and INF-γ. These variables exhibited significant disparities between stroke patients without diabetes and those with comorbid diabetes. Notably, older age correlated positively with the presence of comorbid diabetes in stroke patients (*P* = 0.024, *χ*^2^ = 5.100). Additionally, elevated levels of HDL and IL-2 were positively associated with diabetes in stroke patients (HDL: *P* = 0.004, *χ*^2^ = 9.210; IL-2: *P* = 0.001, *χ*^2^ = 12.313). Conversely, reduced concentrations of CD4, IL-4, IL-6, IL-10, TNF-α, and INF-γ were negatively correlated with diabetes in stroke patients (CD4: *P* = 0.032, *χ*^2^ = 5.281; IL-4: *P* < 0.001, *χ*^2^ = 22.293; IL-6: *P* < 0.001, *χ*^2^ = 22.153; IL-10: *P* < 0.001, *χ*^2^ = 18.807; TNF-α: *P* < 0.001, *χ*^2^ = 23.495; INF-γ: *P* = 0.003, *χ*^2^ = 8.396) (Fig. 2B).

**Table 3:** Connections between diabetes comorbidity and clinical indicators in stroke patients

Variable	Comorbidity		χ^2^	*P*	χ^2^	P*
	No	Yes				
Age (years)						
≥ 60	42	122	5.1	0.024*	/	/
< 60	24	34				
HDL						
1.03–1.55* mg/dl*	32	72			12.106	0.002*
> 1.55* mg/dl*	9	3	9.21	0.004*		
< 1.03* mg/dl*	25	72	0.617	0.432		
CD4						
28.5–60.5%	52	134			5.619	0.06
> 60.5%	6	10	0.079	0.402		
< 28.5%	8	6	5.281	0.032*		
IL-2						
1.1–9.8 pg/ml	54	119			12.307	0.002*
> 9.8 pg/ml	6	0	12.313	0.001*		
< 1.1 pg/ml	6	11	0.119	0.786		
IL-4						
0.1–3 pg/ml	50	107			23.434	< 0.001*
> 3 pg/ml	4	12	0.317	0.778		
< 0.1 pg/ml	12	0	22.293	< 0.001*		
IL-6						
1.7–16.6 pg/ml	52	110			24.007	< 0.001*
> 16.6 pg/ml	2	9	0.929	0.506		
< 1.7 pg/ml	12	0	22.153	< 0.001*		
IL-10						
2.6–4.9 pg/ml	52	110			20.143	< 0.001*
> 4.9 pg/ml	2	8	0.64	0.727		
< 2.6 pg/ml	12	1	18.807	< 0.001*		
TNF-α						
0.1–5.2 pg/ml	52	117			23.767	< 0.001*
> 5.2 pg/ml	2	2	0.673	0.59		
< 0.1 pg/ml	12	0	23.495	< 0.001*		
INF-γ						
1.6–17.3 pg/ml	54	41			11.435	0.003*
> 17.3 pg/ml	0	2	2.565	0.194		
< 1.6 pg/ml	12	0	8.396	0.003*		

More data can be found in Additional file 1: Table S3. *P*: A comparison of different ranges of clinical indicators, and a comparison of clinical indicators that are above or below the normal range with clinical indicators that are within the normal range. P*: Multiple list comparisons between different ranges of clinical indicators

We analyzed the age and 20 clinical indicators of stroke patients with comorbid hyperlipidemia, and the results are documented in Additional file 1: Table S4. Among these indicators, there are 12 differences identified. In Table 4, plasma levels of HDL, IL-2, TG, aPL-IgM, Anti-β2GPI-IgA, Anti-β2GPI-IgM, CD3, IL-4, IL-6, IL-10, TNF-α, and INF-γ were examined for differences between stroke patients with and without comorbid hyperlipidemia. Elevated concentrations of HDL, IL-2, TG, aPL-IgM, Anti-β2GPI-IgA, and Anti-β2GPI-IgM were positively associated with the presence of hyperlipidemia in stroke patients (*P* = 0.022, *χ*^2^ = 5.895; IL-2: *P* = 0.029, *χ*^2^ = 5.597; TG: *P* < 0.001, *χ*^2^ = 28.928; aPL-IgM: *P* = 0.003, *χ*^2^ = 8.565; Anti-β2GPI-IgA: *P* = 0.040, *χ*^2^ = 4.607; Anti -β2GPI-IgM: *P* = 0.002, *χ*^2^ = 9.852). In contrast, lower concentrations of HDL, CD3, IL-4, IL-6, IL-10, TNF-α, and INF-γ were negatively correlated with hyperlipidemia comorbidity in stroke patients (HDL: *P* = 0.048, *χ*^2^ = 4.716; CD3: *P* = 0.003, *χ*^2^ = 9.114; IL-4: *P* = 0.001, *χ*^2^ = 10.633; IL-6: *P* = 0.001, *χ*^2^ = 9.881; IL-10: *P* = 0.020, *χ*^2^ = 6.173; TNF-α: *P* = 0.001, *χ*^2^ = 10.875; INF-γ: *P* = 0.001, *χ*^2^ = 10.717) (Fig. 2C).

**Table 4:** Links between hyperlipidemia comorbidity and clinical indicators in stroke patients

Variable	Comorbidity		χ^2^	*P*	χ^2^	P*
	No	Yes				
LDL						
1.89–4.21 mg*/dl*	52	43			6.182	0.045*
> 4.21* mg/dl*	8	15	2.95	0.106		
< 1.89* mg/dl*	6	1	2.551	0.137		
HDL						
1.03–1.55* mg/dl*	32	43			8.806	0.012*
> 1.55* mg/dl*	9	2	5.895	0.022*		
< 1.03* mg/dl*	25	14	4.716	0.048*		
TG						
≥ 1.70* mg/dl*	12	41	28.928	< 0.001*	/	/
< 1.70* mg/dl*	44	16				
aPL-IgM						
≥ 2.5* mg/dl*	26	34	8.565	0.003*	/	/
< 2.5* mg/dl*	40	17				
Anti-β2GPI-IgA						
≥ 2.0* mg/dl*	6	12	4.607	0.040*	/	/
< 2.0* mg/dl*	60	39				
Anti-β2GPI-IgM						
≥ 2.0* mg/dl*	26	35	9.852	0.002*	/	/
< 2.0* mg/dl*	40	16				
CD3						
59.4–84.6%	45	52			9.308	0.010*
> 84.6%	5	6	0.003	1		
< 59.4%	16	3	9.114	0.003*		
IL-2						
1.1–9.8 pg/ml	54	53	5.597		5.782	0.056
> 9.8 pg/ml	6	0	0.333	0.029*		
< 1.1 pg/ml	6	4		0.744		
IL-4						
0.1–3 pg/ml	50	49			10.718	0.005*
> 3 pg/ml	4	4	0.001	1		
< 0.1 pg/ml	12	0	10.633	0.001*		
IL-6						
1.7–16.6 pg/ml	52	47			12.987	0.002*
> 16.6 pg/ml	2	6	2.243	0.161		
< 1.7 pg/ml	12	0	9.881	0.001*		
IL-10						
2.6–4.9 pg/ml	52	51			7.826	0.020*
> 4.9 pg/ml	2	0	1.926	0.496		
< 2.6 pg/ml	12	2	6.173	0.020*		
TNF-α						
0.1–5.2 pg/ml	52	52			11.045	0.004*
> 5.2 pg/ml	2	1	0.324	1		
< 0.1 pg/ml	12	0	10.875	0.001*		
INF-γ						
1.6–17.3 pg/ml	54	53			10.717	0.001*
> 17.3 pg/ml	0	0	/	/		
< 1.6 pg/ml	12	0	10.717	0.001*		

More data can be found in Additional file 1: Table S4. *P*: A comparison of different ranges of clinical indicators, and a comparison of clinical indicators that are above or below the normal range with clinical indicators that are within the normal range. P*: Multiple list comparisons between different ranges of clinical indicators

We conducted an analysis of the age and 20 clinical indicators of stroke patients with comorbid atrial fibrillation, and the findings are outlined in Additional file 1: Table S5. Among these indicators, 8 distinct differences were observed. Table 5 reveals significant distinctions in the age of patients and plasma levels of LDL, HDL, IL-4, IL-6, IL-10, TNF-α, and INF-γ between stroke patients without atrial fibrillation and those with comorbid atrial fibrillation. Older age exhibited a positive correlation with atrial fibrillation in stroke patients (*P* < 0.001, *χ*^2^ = 12.141). Furthermore, reduced levels of LDL, HDL, IL-4, IL-6, IL-10, TNF-α, and INF-γ were negatively correlated with atrial fibrillation comorbidity in stroke patients (LDL: *P* = 0.006, *χ*^2^ = 8.250; HDL: *P* = 0.034, *χ*^2^ = 4.496; IL-4: *P* = 0.007, *χ*^2^ = 6.889; IL-6: *P* = 0.007, *χ*^2^ = 6.857; IL-10: *P* = 0.031, *χ*^2^ = 4.823; TNF-α: *P* = 0.007, *χ*^2^ = 7.468; INF-γ: *P* = 0.007, *χ*^2^ = 7.222) (Fig. 2D).

**Table 5:** Associations between atrial fibrillation comorbidity and clinical indicators in stroke patients

Variable	Comorbidity		χ^2^	*P*	χ^2^	P*
	No	Yes				
Age (years)						
≥ 60	42	45	12.141	< 0.001*	/	/
< 60	24	4				
LDL						
1.89–4.21 mg/dl	52	30			8.772	0.012*
> 4.21 mg/dl	8	4	0.048	1		
< 1.89 mg/dl	6	15	8.25	0.006*		
HDL						
1.03–1.55 mg/dl	32	17			9.325	0.009*
> 1.55 mg/dl	9	1	2.389	0.154		
< 1.03 mg/dl	25	31	4.496	0.034*		
IL-4						
0.1–3 pg/ml	50	31			8.478	0.014*
> 3 pg/ml	4	5	1.008	0.475		
< 0.1 pg/ml	12	0	6.889	0.007*		
IL-6						
1.7–16.6 pg/ml	52	32			9.42	0.009*
> 16.6 pg/ml	2	4	1.905	0.213		
< 1.7 pg/ml	12	0	6.857	0.007*		
IL-10						
2.6–4.9 pg/ml	52	33			5.179	0.075
> 4.9 pg/ml	2	2	0.2	0.644		
< 2.6 pg/ml	12	1	4.823	0.031*		
TNF-α						
0.1–5.2 pg/ml	52	35			7.479	0.024*
> 5.2 pg/ml	2	1	0.057	1		
< 0.1 pg/ml	12	0	7.468	0.007*		
INF-γ						
1.6–17.3 pg/ml	54	35	1.517	0.4	9.012	0.011*
> 17.3 pg/ml	0	1				
< 1.6 pg/ml	12	0	7.222	0.007*		

More data can be found in Additional file 1: Table S5. *P*: A comparison of different ranges of clinical indicators, and a comparison of clinical indicators that are above or below the normal range with clinical indicators that are within the normal range. P*: Multiple list comparisons between different ranges of clinical indicators

We studied the age and 20 clinical indicators of stroke patients with comorbid hyperhomocysteinemia, and the results are presented in Additional file 1: Table S6. Among these indicators, three differences were identified. Comparatively, Table 6 indicates that the number of stroke patients with hyperhomocysteinemia exhibited no significant differences in age, blood lipid indicators, anticardiolipin antibodies, and lymphocyte subpopulations when compared to those without the condition. However, significant differences were observed in Th1/Th2 lymphocyte cytokine detection for patients with comorbid hyperhomocysteinemic stroke. Lower concentrations of IL-4, IL-6, and TNF-α were negatively correlated with stroke patients suffering from hyperhomocysteinemia (IL-4: *P* = 0.015, *χ*^2^ = 6.040; IL-6: *P* = 0.015, *χ*^2^ = 6.038; TNF-α: *P* = 0.016, *χ*^2^ = 6.243) (Fig. 2E).

**Table 6:** Correlations between hyperhomocysteinemia comorbidity and clinical indicators in stroke patients

Variable	Comorbidity		χ^2^	*P*	χ^2^	P*
	No	Yes				
IL-4						
0.1–3 pg/ml	50	27			6.043	0.049*
> 3 pg/ml	4	2	0.007	1		
< 0.1 pg/ml	12	0	6.04	0.015*		
IL-6						
1.7–16.6 pg/ml	52	28			6.039	0.049*
> 16.6 pg/ml	2	1	0.004	1		
< 1.7 pg/ml	12	0	6.038	0.015*		
TNF-α						
0.1–5.2 pg/ml	52	29			7.215	0.027*
> 5.2 pg/ml	2	0	1.101	0.54		
< 0.1 pg/ml	12	0	6.243	0.016*		

More data can be found in Additional file 1: Table S6*. P*: A comparison of different ranges of clinical indicators, and a comparison of clinical indicators that are above or below the normal range with clinical indicators that are within the normal range. P*: Multiple list comparisons between different ranges of clinical indicators

### The synergistic effect of multiple risk factors

In Table 1, we classified stroke patients into two groups based on the number of risk factors they presented: those without any risk factors (*n* = 66) and those with multiple risk factors (*n* = 371). Using stroke patients without any risk factors as the control group, we proceeded to analyze the differences and correlations between varying concentration ranges of clinical indicators in cases of stroke patients with multiple risk factors (Fig. 1).

We analyzed the age and 20 clinical indicators of stroke patients with multiple diseases, and the findings are displayed in Additional file 1: Table S7. Among these indicators, six differences were observed. Table 7 highlights the levels of IL-2, IL-4, IL-6, IL-10, TNF-α, and INF-γ in plasma, demonstrating significant differences between stroke patients without comorbid diseases and stroke patients with multiple risk factors. Particularly, heightened levels of IL-2 were positively correlated with the presence of multiple risk factors in stroke patients (*P* < 0.001, *χ*^2^ = 27.701). Conversely, decreased concentrations of IL-4, IL-6, IL-10, TNF-α, and INF-γ were negatively correlated with stroke patients suffering from multiple risk factors (IL-4: *P* < 0.001, *χ*^2^ = 49.153; IL-6: *P* < 0.001, *χ*^2^ = 50.059; IL-10: *P* < 0.001, *χ*^2^ = 32.126; TNF-α: *P* < 0.001, *χ*^2^ = 51.946; INF-γ: *P* < 0.001, *χ*^2^ = 50.909) (Fig. 2F).

**Table 7:** Interactions between multiple risk factors and clinical indicators in stroke patients

Variable	Comorbidity		χ^2^	*P*	χ^2^	P*
	No	Yes				
HDL						
1.03–1.55* mg/dl*	32	198			7.604	0.022*
> 1.55* mg/dl*	9	17	7.443	0.02		
< 1.03* mg/dl*	25	139	0.137	0.936		
IL-2						
1.1–9.8 pg/ml	54	272			27.826	< 0.001*
> 9.8 pg/ml	6	0	27.701	< 0.001*		
< 1.1 pg/ml	6	29	0.008	1		
IL-4						
0.1–3 pg/ml	50	244			51.648	< 0.001*
> 3 pg/ml	4	28	0.424	0.624		
< 0.1 pg/ml	12	0	49.153	< 0.001*		
IL-6						
1.7–16.6 pg/ml	52	257			51.538	< 0.001*
> 16.6 pg/ml	2	15	0.299	0.748		
< 1.7 pg/ml	12	0	50.059	< 0.001*		
IL-10						
2.6–4.9 pg/ml	52	254			33.086	< 0.001*
> 4.9 pg/ml	2	14	0.22	1		
< 2.6 pg/ml	12	4	32.126	< 0.001*		
TNF-α						
0.1–5.2 pg/ml	52	267			51.931	< 0.001*
> 5.2 pg/ml	2	5	0.746	0.327		
< 0.1 pg/ml	12	0	51.946	< 0.001*		
INF-γ						
1.6–17.3 pg/ml	54	270			51.626	< 0.001*
> 17.3 pg/ml	0	2	0.4	1		
< 1.6 pg/ml	12	0	50.909	< 0.001*		

More data can be found in Additional file 1: Table S7*. P*: A comparison of different ranges of clinical indicators, and a comparison of clinical indicators that are above or below the normal range with clinical indicators that are within the normal range. P*: Multiple list comparisons between different ranges of clinical indicators

## Changes in clinical indicators across various ischemic stroke subtypes

Stroke patients were stratified into non-ischemic stroke (*n* = 303) and ischemic stroke (*n* = 118) groups based on the TOAST (Trial of Org 10172 in Acute Stroke Treatment) definition [27], as outlined in Table 1. Within the ischemic stroke category, three predominant subtypes were identified: lacunar infarction (*n* = 87), cardioembolic embolism (*n* = 7), and large artery atherosclerosis (*n* = 24). A comparative analysis was conducted, utilizing non-ischemic stroke patients as the reference group. The study specifically examined differences and correlations in clinical indicators across various concentration ranges when patients experienced different ischemic stroke subtypes (As shown in Table 1 and Fig. 1).

**Fig. 3:**
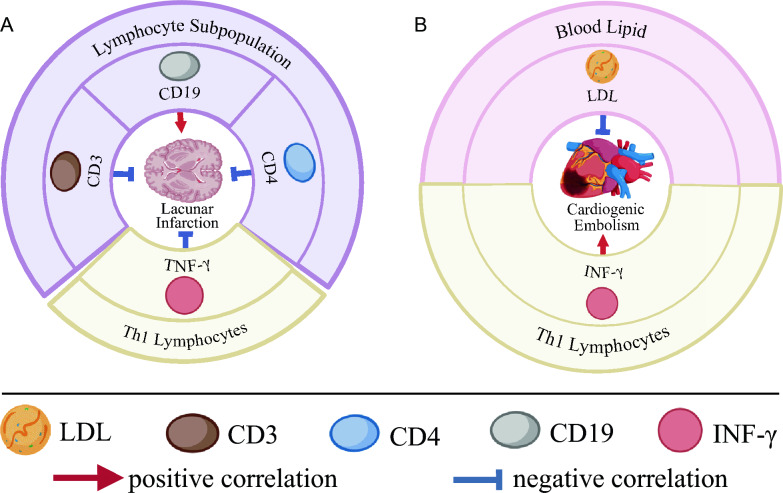
Correlation analysis between subtypes of ischemic stroke and clinical indicators. **A** Lacunar Infarction. Positively correlated: CD19; Negatively correlated: CD3, CD4, INF-γ. **B** Cardiogenic Embolism. Positively related: INF-γ; Negatively correlated: LDL

As illustrated in Table 8, notable disparities in plasma levels of CD3, CD4, CD19, and INF-γ emerge when comparing ischemic stroke subtypes, specifically lacunar infarction, with non-ischemic stroke subtypes. Remarkably, elevated concentrations of CD19 exhibit a positive correlation with lacunar infarction (*P* = 0.241, *χ*^2^ = 4.854). Conversely, diminished concentrations of CD3, CD4, and INF-γ demonstrate negative correlations with lacunar infarction (CD3: *P* = 0.004, χ^2^ = 8.184; CD4: *P* = 0.009, *χ*^2^ = 6.396; INF-γ: *P* = 0.003, *χ*^2^ = 13.132) (Fig. 3A).

**Table 8:** The risk of clinical indicators in stroke patients with lacunar infarction

Variable	Stroke Subtypes		*χ*^*2*^	*P*_1_	*χ*^*2*^	*P*_2_
	No	Yes				
CD3						
59.4–84.6%	241	69			9.028	0.011*
> 84.6%	15	6	0.447	0.589		
< 59.4%	58	4	8.184	0.004*		
CD4						
28.5–60.5%	266	80			7.272	0.026*
> 60.5%	19	3	1.065	0.432		
< 28.5%	29	1	6.396	0.009*		
CD19						
6.4–22.4%	232	72			5.205	0.074
> 22.4%	29	2	4.854	0.024*		
< 6.4%	43	10	0.592	0.442		
INF-γ						
1.6–17.3 pg/ml	230	67			13.471	0.001*
> 17.3 pg/ml	1	0	0.291	1.000		
< 1.6 pg/ml	0	4	13.132	0.003*		

More data can be found in Additional file 1: Table S6*. P*: A comparison of different ranges of clinical indicators, and a comparison of clinical indicators that are above or below the normal range with clinical indicators that are within the normal range. P*: Multiple list comparisons between different ranges of clinical indicators

As depicted in Table 9, noteworthy distinctions in plasma LDL and INF-γ levels emerge among ischemic stroke subtypes, namely cardioembolism and non-ischemic stroke subtypes. Notably, elevated concentrations of INF-γ exhibit a positive correlation with cardioembolic disease (*P* = 0.050, *χ*^2^ = 18.418). Conversely, diminished concentrations of LDL demonstrate an inverse association with cardioembolic disease (LDL: *P* = 0.027, *χ*^2^ = 7.920) (Fig. 3B).

**Table 9:** The risk of clinical indicators in stroke patients with cardiogenic embolism

Variable	Stroke subtypes		*χ*^*2*^	*P*_1_	*χ*^*2*^	*P*_2_
	No	Yes				
LDL						
1.89–4.21 mg*/dl*	250	3			7.935	0.019*
> 4.21* mg/dl*	20	1	1.724	0.274		
< 1.89* mg/dl*	33	3	7.920	0.027*		
IL-10						
2.6–4.9 pg/ml	216	5			11.937	0.003*
> 4.9 pg/ml	13	0	0.301	1.000		
< 2.6 pg/ml	2	1	10.961	0.079		
INF-γ						
1.6–17.3 pg/ml	230	5			18.418	0.050*
> 17.3 pg/ml	1	1	18.418	0.050*		
< 1.6 pg/ml	0	0	/	/		

More data can be found in Additional file 1: Table S6*. P*: A comparison of different ranges of clinical indicators, and a comparison of clinical indicators that are above or below the normal range with clinical indicators that are within the normal range. P*: Multiple list comparisons between different ranges of clinical indicators

## Discussion

Hypertension constitutes an autonomous risk factor for ischemic stroke, a well-established assertion supported by substantial evidence [29]. Of notable significance, a considerable body of research underscores the substantive association between plasma levels of LDL and HDL and the heightened risk of hypertension, substantiated across multiple independent studies [30–33]. Our research, likewise, substantiates these findings by demonstrating that stroke patients with concurrent hypertension exhibit significantly elevated levels of both LDL and HDL in their plasma—a consistent alignment with prior investigations. Moreover, our study identifies a noteworthy connection between diminished plasma CD19 levels and the presence of comorbid hypertension. This observed correlation suggests a possible link to immune system disturbances induced by elevated blood pressure, with support from an analogous study, which registered a declining trend in CD19 gene expression among hypertension patients [34].

Diabetes, as an autonomous risk factor for stroke, is intricately intertwined with advancing age, a relationship underscored by a meticulously conducted study [9]. This comprehensive analysis highlights a conspicuous association between increasing age and the co-occurrence of diabetes among stroke-afflicted individuals—an observation harmonizing with antecedent research. Moreover, the study by Sun et al. posits advancing age as a predisposing factor for diabetes [35]. Intriguingly, our investigation unveils an additional dimension: significantly elevated HDL levels among stroke patients concurrently affected by diabetes, a finding diverging from Parhofer et al.’s postulation that diminished HDL levels serve as a hallmark of diabetic dyslipidemia [36].

Hyperlipidemia, recognized as a prominent risk factor for atherosclerosis—a leading cause of stroke—is inherently characterized by elevations in plasma LDL and TG levels, concomitant with a reduction in high-density lipoprotein levels [37]. Our study contributes to the existing body of knowledge by establishing a compelling association between fluctuations in plasma HDL levels and the co-occurrence of hyperlipidemia in stroke patients. Furthermore, we ascertain that diminished levels of aPL-IgM, Anti-β2GPI-IgA, and Anti-β2GPI-IgM exhibit a noteworthy association with comorbid hyperlipidemia in stroke patients. These findings are in alignment with established research, affirming that hyperlipidemia precipitates an elevation in LDL, a diminution in HDL, and an increase in anticardiolipin antibodies in plasma, thereby escalating the risk of atherosclerosis and thrombosis [38–40].

Atrial fibrillation, a prevalent cardiac arrhythmia, exhibits a discernible correlation with age-based incidence [41]. Research endeavors have delineated the intricate interplay between LDL and atrial fibrillation, with select studies suggesting an inverse relationship, implying that decreased LDL levels may trigger atrial fibrillation onset [42, 43]. Paradoxically, an alternative study hints at the prospect of diminished HDL levels augmenting the susceptibility to atrial fibrillation [44]. In our investigation, we ascertained that reduced plasma LDL and HDL levels in stroke patients are significantly linked with comorbid atrial fibrillation, a concurrence consistent with antecedent research. Moreover, extant studies substantiate the interconnectedness of LDL, HDL, and atherosclerosis [30, 45], with atherosclerosis recognized as a known precipitant of atrial fibrillation [46, 47].

The equilibrium of Th1/Th2 cytokines holds paramount importance in the genesis and progression of stroke, wherein post-stroke imbalances tend to favor Th1-type responses—inclusive of IFN-γ, TNF-α, and IL-2—potentiating the exacerbation of cerebral damage and neurologic deficits [48]. In contrast to precedent investigations, our research unearthed a distinctive immune response among stroke patients grappling with one or more risk factors, characterized by diminished levels of various helper T cell 1 (Th1)/Th2 factors in their plasma, except for elevated IL-2 levels. This unique immune response warrants comprehensive exploration to elucidate the underlying mechanisms at play.

Anticardiolipin antibodies, a category of autoantibodies directed against cardiolipin or its derivatives, encompass aPL-IgM, Anti-β2GPI-IgA, and Anti-β2GPI-IgM [49]. Notably, antiphospholipid syndrome has been identified as a contributory factor in acute ischemic stroke and transient ischemic attacks [40]. Elevated levels of anticardiolipin antibodies render the blood hypercoagulable, thereby amplifying the risk of thrombosis [38, 39]. Lymphocyte subsets represent a pivotal component of the immune system, actively participating in inflammatory responses and adaptive immunity. Inflammation holds a significant role in the genesis and progression of stroke, precipitating vascular endothelial damage, vascular remodeling, and atherosclerosis, collectively heightening blood pressure and cardiovascular risk [50]. Th1/Th2 cells serve as key modulators of immune and inflammatory responses. Cytokines produced by Th1 cells, including IFN-γ, TNF-α, and IL-2, elicit pro-inflammatory and cytotoxic effects. Conversely, cytokines stemming from Th2 cells, such as IL-4, IL-6, and IL-10, convey anti-inflammatory and immunomodulatory properties, counteracting the pro-inflammatory response of Th1 [51]. A noteworthy observation is the altered balance of Th1/Th2 lymphocyte cytokines post-stroke, leading to a preponderance of Th1-type responses, thereby exacerbating cerebral damage and neurologic deficits [48].

Reviewing prior studies, it becomes apparent that there exists a paucity of systematic analyses elucidating the disparities among stroke risk factors concerning four distinct indicators: lymphocyte subpopulations, Th1/Th2 lymphocyte cytokines, blood lipid markers, and antiphospholipid antibody parameters. Our study bridges this gap, offering valuable insights into the distinct clinical indicators when stroke patients grapple with multiple risk factors. Our findings underscore that divergent clinical indicators exhibit increased prominence when multiple risk factors are concurrent in stroke patients. A comprehensive literature search revealed a dearth of articles investigating the clinical indicators and underlying reasons for the elevated stroke risk when patients present with multiple risk factors. It is our aspiration that researchers will draw inspiration from our study to address these gaps in knowledge. Simultaneously, our study underscores the imperative of considering not only the individual role of a singular risk factor but also the synergistic effects that arise when multiple risk factors converge in the context of stroke prevention and treatment.

Ischemic stroke, comprising lacunar infarction, cardioembolic, and large artery atherosclerosis subtypes, represents a significant medical challenge [28]. T lymphocytes play a pivotal role in the pathophysiology of ischemic stroke, regulating the immune response and inflammatory processes in the body [52]. A comprehensive investigation into the dynamic changes of T lymphocytes following ischemic stroke revealed an increase in peripheral T lymphocyte numbers post-event [53].

Examining patients diagnosed with lacunar infarcts, our study identified a higher prevalence of elevated CD3 and CD4 concentrations compared to lower concentrations, aligning with existing research. In contrast, lower concentrations of CD19 affected a larger proportion of individuals than higher concentrations, contradicting prior studies. Literature reports suggest a positive correlation between elevated Th1 cells and acute ischemic stroke [54]. Similarly, within the cardioembolic stroke subtype, our findings indicated a higher incidence of elevated INF-γ concentrations, consistent with previous research. Conversely, within the lacunar infarct subtype, a higher prevalence of lower INF-γ concentrations was observed, contrary to earlier findings.

Furthermore, our investigation within the cardioembolic stroke subtypes unveiled a greater impact of lower concentrations of LDL on a larger population than higher concentrations. Notably, some studies have associated lower LDL levels with an increased risk of hemorrhagic stroke [55], corroborating our findings. However, a Mendelian randomization study exploring the impact of LDL levels on ischemic stroke subtypes revealed a smaller effect on cardioembolic strokes compared to lacunar infarction and large artery atherosclerosis subtypes [56], contradicting our observations. These inconsistencies highlight the complex and multifaceted nature of ischemic stroke and warrant further exploration to refine our understanding of its underlying mechanisms.

### Study limitations

Several limitations warrant acknowledgment. First, our study's sample size remained relatively small, and the cohort exclusively emanated from the First People's Hospital of Wenling City, Zhejiang Province, China, potentially limiting its representativeness and external generalizability. Expanding the sample size and geographical scope is essential to validate the robustness of these findings. Second, our methodology did not exclude patients with overlapping risk factors when calculating stroke incidence in patients with individual risk factors. This omission may affect the precision of our results, albeit without undermining the overarching conclusions. Third, the study did not account for potential medication usage among patients, a factor that could introduce variance in the results. Our study faced limitations stemming from an insufficient amount of sample data, hindering our ability to delve into the risk associated with clinical indicators among stroke patients grappling with three or more risk factors. Simultaneously, the scope of our investigation was constrained, preventing an in-depth exploration of the variations in clinical indicators among individuals aged 85 and above afflicted with ischemic stroke subtypes, particularly when confronted with diverse coexisting risk factors.

## Conclusions

In summary, our study arrives at several noteworthy conclusions. Firstly, advancing age corresponds with an elevated risk of stroke. Secondly, aberrations in blood lipids and Th1/Th2 cytokines emerge as common denominators among multiple risk factors for stroke, playing pivotal roles in the initiation and progression of this cerebrovascular condition. Lastly, an array of inflammatory responses, particularly those triggered by atherosclerosis, actively contributes to the onset and development of stroke, ultimately influencing prognosis. Examining alterations in clinical indicators among elderly stroke patients aged 85 and above, under the influence of various risk factors, proves instructive. This investigation holds substantial significance for enhancing the understanding of treatment modalities and prognostic outcomes in the context of elderly stroke patients.

### Supplementary Information

**Additional file 1: Table S2.** The risk of clinical indicators in stroke patients with comorbid hypertension. **Table S3.** The risk of clinical indicators in stroke patients with comorbid diabetes mellitus. **Table S4.** The risk of clinical indicators in stroke patients with comorbid hyperlipidemia. **Table S5.** The risk of clinical indicators in stroke patients with comorbid atrialfibrillation. **Table S6.** The risk of clinical indicators in stroke patients with comorbid hyperhomocysteinaemia. **Table S7.** The risk of clinical indicators in stroke patients with comorbid multiple disease. **Table S8.** The risk of clinical indicators in stroke patients with lacunar infarction. **Table S9.** The risk of clinical indicators in stroke patients with cardiogenic embolism. **Table S10.** The risk of clinical indicators in stroke patients with aorta atherosclerosis.

## Data Availability

The datasets generated and/or analyzed during the current study are not publicly available due data are not public but are available from the corresponding author on reasonable request.

## Abbreviations

AF: Atrial fibrillation
Anti-β2GPI-IgA: Antibodies against β2 Glycoprotein I-IgA
Anti-β2GPI-IgG: Antibodies against β2 Glycoprotein I-IgG
Anti-β2GPI-IgM: Antibodies against β2 Glycoprotein I-IgM
aPL-IgA: Antiphospholipid antibodies-IgA
aPL-IgG: Antiphospholipid antibodies-IgG
aPL-IgM: Antiphospholipid antibodies-IgM
CD19: CD19 is a protein that is expressed on the surface of B cells
CD3: Cluster of differentiation 3
CD4: Helper T cell 4
CD4/8: The ratio of CD4 T cell to CD8 T cell
CD8: Cytotoxic T cell
DM: Diabetes mellitus
Hcy: Homocysteine
HDL: High-density lipoprotein
HHcy: Hyperhomocysteinemia
HL: Hyperlipidemia
HTN: Hypertension
IFN-γ: Interferon-γ
IL-10: Interleukin-10
IL-2: Interleukin-2
IL-4: Interleukin-4
IL-6: Interleukin-6
LDL: Low-density lipoprotein
NK: Natural killer cells
TG: Triglycerides
TNF-α: Tumor necrosis factor-α
