# Supplementary material for: Exploring clinical indicator variations in stroke patients with multiple risk factors: focus on hypertension and inflammatory reactions
Source: Eur J Med Res. 2024 Jan 29;29:81. doi: 10.1186/s40001-024-01653-6 (PMC10823715; doi:10.1186/s40001-024-01653-6)
Supplement: Supplementary file 1 — Additional file 1: Table S2. The risk of clinical indicators in stroke patients with comorbid hypertension. Table S3. The risk of clinical indicators in stroke patients with comorbid diabetes mellitus. Table S4. The risk of clinical indicators in stroke patients with comorbid hyperlipidemia. Table S5. The risk of clinical indicators in stroke patients with comorbid atrialfibrillation. Table S6. The risk of clinical indicators in stroke patients with comorbid hyperhomocysteinaemia. Table S7. The risk of clinical indicators in stroke patients with comorbid multiple disease. Table S8. The risk of clinical indicators in stroke patients with lacunar infarction. Table S9. The risk of clinical indicators in stroke patients with cardiogenic embolism. Table S10. The risk of clinical indicators in stroke patients with aorta atherosclerosis. [file 40001_2024_1653_MOESM1_ESM.htm]

**Additional
Form**

**Table
S2. The risk of clinical
indicators in stroke patients with comorbid hypertension.**

|  |  |  |  |  |  |  |  |
| --- | --- | --- | --- | --- | --- | --- | --- |
| **Variable** | **Comorbidity** | | ***¦Ö*****2** | ***P*** | | ***¦Ö*****2** | ***P\**** |
|  | **No** | **Yes** |  |  |  |  |  |
| **Age (years)** | ¡¡ | | | | | | |
| ¡Ý60 | 42 | 214 | 2.074 | 0.15 | | / | / |
| £¼60 | 24 | 81 |  |  |  |  |  |
| **Blood Lipid Profile** | | | | | | | |
| **LDL** |  | | | | | | |
| 1.89-4.21*mg/dl* | 52 | 237 |  |  | | 5.428 | 0.066 |
| £¾4.21*mg/dl* | 8 | 13 | 5.068 | **0.040\*** | |  |  |
| £¼1.89*mg/dl* | 6 | 32 | 0.112 | 0.825 | |  |  |
| **HDL** |  | | | | | | |
| 1.03-1.55 *mg/dl* | 32 | 162 |  | |  | 7.622 | **0.022\*** |
| £¾1.55*mg/dl* | 9 | 13 | 7.658 | | **0.010\*** |  |  |
| £¼1.03*mg/dl* | 25 | 93 | 1.082 | | 0.365 |  |  |
| **TG** |  | | | | | | |
| ¡Ý1.70 *mg/dl* | 12 | 77 | 0.832 | 0.362 | | / | / |
| <1.70 *mg/dl* | 44 | 205 |  |  |  |  |  |
| **Antiphospholipid Antibody** | | | | | | | |
| **aPL-IgA** |  | | | | | | |
| ¡Ý2.5 *mg/dl* | 6 | 16 | 0.362 | 0.594 | | / | / |
| £¼2.5*mg/dl* | 60 | 216 |  |  |  |  |  |
| **aPL-IgG** |  | | | | | | |
| ¡Ý2.0 *mg/dl* | 20 | 63 | 0.234 | 0.629 | | / | / |
| £¼2.0*mg/dl* | 46 | 168 |  |  |  |  |  |
| **aPL-IgM** |  | | | | | | |
| ¡Ý2.5 *mg/dl* | 26 | 106 | 0.877 | 0.349 | | / | / |
| £¼2.5 *mg/dl* | 40 | 125 |  |  |  |  |  |
| **Anti-¦Â2GPI-IgA** |  | | | | | | |
| ¡Ý2.0 *mg/dl* | 6 | 33 | 1.214 | 0.309 | | / | / |
| £¼2.0 *mg/dl* | 60 | 198 |  |  |  |  |  |
| **Anti-¦Â2GPI-IgG** |  | | | | | | |
| ¡Ý2.0 *mg/dl* | 19 | 67 | 0.001 | 0.973 | | / | / |
| £¼2.0 *mg/dl* | 47 | 164 |  |  |  |  |  |
| **Anti-¦Â2GPI-IgM** |  | | | | | | |
| ¡Ý2.0 *mg/dl* | 26 | 97 | 2.619 | 0.706 | | / | / |
| £¼2.0 *mg/dl* | 40 | 134 |  |  |  |  |  |
| **Lymphocyte Subpopulation** | | | | | | | |
| **CD3** |  | | | | | | |
| 59.4-84.6% | 45 | 223 |  |  | | 1.564 | 0.458 |
| £¾84.6% | 5 | 18 | 0.364 | 0.565 | |  |  |
| £¼59.4% | 16 | 54 | 1.381 | 0.24 | |  |  |
| **CD4** |  | | | | | | |
| 28.5-60.5% | 52 | 250 |  |  | | 3.281 | 0.194 |
| £¾60.5% | 6 | 15 | 1.718 | 0.235 | |  |  |
| £¼28.5% | 8 | 21 | 1.916 | 0.204 | |  |  |
| **CD8** |  | | | | | | |
| 11.1-38.3% | 56 | 260 |  |  | | 2.145 | 0.342 |
| £¾38.3% | 5 | 13 | 1.154 | 0.342 | |  |  |
| £¼11.1% | 5 | 13 | 1.154 | 0.342 | |  |  |
| **CD4/8** |  | | | | | | |
| 1.1-2.5% | 36 | 157 |  |  | | 0.009 | 0.995 |
| £¾2.5% | 23 | 98 | 0.006 | 0.937 | |  |  |
| £¼1.1% | 7 | 31 | 0.001 | 1 | |  |  |
| **CD19** |  | | | | | | |
| 6.4-22.4% | 44 | 226 |  |  | | 5.34 | 0.069 |
| £¾22.4% | 7 | 19 | 1.878 | 0.272 | |  |  |
| £¼6.4% | 15 | 38 | 4.277 | **0.039\*** | |  |  |
| **NK** |  | | | | | | |
| 5.6-30.9% | 49 | 227 |  |  | | 3.462 | 0.177 |
| £¾30.9% | 7 | 33 | 0.002 | 0.969 | |  |  |
| £¼5.6% | 10 | 22 | 3.373 | 0.093 | |  |  |
| **Th1/Th2 Lymphocytes** | | | | | | | |
| **IL-2** |  | | | | | | |
| 1.1-9.8pg/ml | 54 | 214 |  |  | | 21.985 | **<0.001\*** |
| £¾9.8 pg/ml | 6 | 0 | 21.879 | **<0.001\*** | |  |  |
| £¼1.1 pg/ml | 6 | 23 | 0.005 | 1 | |  |  |
| **IL-4** |  | | | | | | |
| 0.1-3 pg/ml | 50 | 193 |  |  | | 40.915 | **<0.001\*** |
| £¾3 pg/ml | 4 | 21 | 0.295 | 0.794 | |  |  |
| £¼0.1 pg/ml | 12 | 0 | 39.2 | **<0.001\*** | |  |  |
| **IL-6** |  | | | | | | |
| 1.7-16.6 pg/ml | 52 | 206 |  |  | | 40.651 | **<0.001\*** |
| £¾16.6 pg/ml | 2 | 8 | 0 | 1 | |  |  |
| £¼1.7 pg/ml | 12 | 0 | 40.422 | **<0.001\*** | |  |  |
| **IL-10** |  | | | | | | |
| 2.6-4.9 pg/ml | 52 | 200 |  |  | | 40.58 | **<0.001\*** |
| £¾4.9 pg/ml | 2 | 12 | 0.33 | 0.742 | |  |  |
| £¼2.6v | 12 | 2 | 30.744 | **<0.001\*** | |  |  |
| **TNF-¦Á** |  | | | | | | |
| 0.1-5.2 pg/ml | 52 | 210 |  |  | | 41.243 | **<0.001\*** |
| £¾5.2 pg/ml | 2 | 4 | 0.663 | 0.348 | |  |  |
| £¼0.1 pg/ml | 12 | 0 | 41.178 | **<0.001\*** | |  |  |
| **INF-¦Ã** |  | | | | | | |
| 1.6-17.3 pg/ml | 54 | 213 |  |  | | 40.877 | **<0.001\*** |
| £¾17.3 pg/ml | 0 | 1 | 0.253 | 1 | |  |  |
| £¼1.6 pg/ml | 12 | 0 | 40.468 | **<0.001\*** | |  |  |
|  |  |  |  |  |  |  |  |

*P*:
A comparison of different ranges of clinical indicators, and a comparison of
clinical indicators that are above or below the normal range with clinical
indicators that are within the normal range. *P*\*: Multiple list
comparisons between different ranges of clinical indicators.

  

**Table
S3. The risk of clinical
indicators in stroke patients with comorbid diabetes****mellitus.**

|  |  |  |  |  |  |  |
| --- | --- | --- | --- | --- | --- | --- |
| **Variable** | **Comorbidity** | | **¦Ö2** | ***P*** | **¦Ö2** | ***P\**** |
|  | **No** | **Yes** |  |  |  |  |
| **Age (years)** |  | | | | | |
| ¡Ý60 | 42 | 122 | 5.1 | **0.024\*** | / | / |
| £¼60 | 24 | 34 |  |  |  |  |
| **Blood Lipid Profile** | | | | | | |
| **LDL** |  | | | | | |
| 1.89-4.21*mg/dl* | 52 | 112 |  |  | 2.237 | 0.327 |
| £¾4.21 *mg/dl* | 8 | 12 | 0.558 | 0.458 |  |  |
| £¼1.89 *mg/dl* | 6 | 23 | 1.423 | 0.277 |  |  |
| **HDL** |  | | | | | |
| 1.03-1.55 *mg/dl* | 32 | 72 |  |  | 12.106 | **0.002\*** |
| £¾1.55 *mg/dl* | 9 | 3 | 9.21 | **0.004\*** |  |  |
| £¼1.03 *mg/dl* | 25 | 72 | 0.617 | 0.432 |  |  |
| **TG** |  | | | | | |
| ¡Ý1.70 *mg/dl* | 12 | 50 | 3.028 | 0.082 | / | / |
| <1.70 *mg/dl* | 44 | 97 |  |  |  |  |
| **Antiphospholipid Antibody** | | | | | | |
| **aPL-IgA** |  | | | | | |
| ¡Ý2.5 *mg/dl* | 6 | 12 | 0.085 | 1 | / | / |
| £¼2.5 *mg/dl* | 60 | 103 |  |  |  |  |
| **aPL-IgG** |  | | | | | |
| ¡Ý2.0 *mg/dl* | 20 | 35 | 0 | 0.985 | / | / |
| £¼2.0 *mg/dl* | 46 | 80 |  |  |  |  |
| **aPL-IgM** |  | | | | | |
| ¡Ý2.5 *mg/dl* | 26 | 55 | 1.206 | 0.272 | / | / |
| £¼2.5 *mg/dl* | 40 | 60 |  |  |  |  |
| **Anti-¦Â2GPI-IgA** |  | | | | | |
| ¡Ý2.0 *mg/dl* | 6 | 19 | 1.945 | 0.186 | / | / |
| £¼2.0 *mg/dl* | 60 | 96 |  |  |  |  |
| **Anti-¦Â2GPI-IgG** |  |  |  |  |  |  |
| ¡Ý2.0 *mg/dl* | 19 | 41 | 0.892 | 0.345 | / | / |
| £¼2.0 *mg/dl* | 47 | 74 |  |  |  |  |
| **Anti-¦Â2GPI-IgM** |  | | | | | |
| ¡Ý2.0 *mg/dl* | 26 | 55 | 1.206 | 0.272 | / | / |
| £¼2.0 *mg/dl* | 40 | 60 |  |  |  |  |
| **Lymphocyte Subpopulation** | | | | | | |
| **CD3** |  | | | | | |
| 59.4-84.6% | 45 | 123 |  |  | 3.5 | 0.174 |
| £¾84.6% | 5 | 11 | 0.147 | 0.77 |  |  |
| £¼59.4% | 16 | 22 | 3.489 | 0.077 |  |  |
| **CD4** |  | | | | | |
| 28.5-60.5% | 52 | 134 |  |  | 5.619 | 0.06 |
| £¾60.5% | 6 | 10 | 0.079 | 0.402 |  |  |
| £¼28.5% | 8 | 6 | 5.281 | **0.032\*** |  |  |
| **CD8** |  | | | | | |
| 11.1-38.3% | 56 | 139 |  |  | 3.659 | 0.16 |
| £¾38.3% | 5 | 7 | 0.912 | 0.342 |  |  |
| £¼11.1% | 5 | 4 | 2.956 | 0.131 |  |  |
| **CD4/8** |  | | | | | |
| 1.1-2.5% | 36 | 85 |  |  | 0.084 | 0.959 |
| £¾2.5% | 23 | 50 | 0.066 | 0.797 |  |  |
| £¼1.1% | 7 | 15 | 0.038 | 0.806 |  |  |
| **CD19** |  | | | | | |
| 6.4-22.4% | 44 | 117 |  |  | 4.397 | 0.111 |
| £¾22.4% | 7 | 13 | 0.517 | 0.444 |  |  |
| £¼6.4% | 15 | 18 | 4.251 | 0.06 |  |  |
| **NK** |  | | | | | |
| 5.6-30.9% | 49 | 122 |  |  | 2.24 | 0.326 |
| £¾30.9% | 7 | 11 | 0.818 | 0.418 |  |  |
| £¼5.6% | 10 | 14 | 1.689 | 0.236 |  |  |
| **Th1/Th2 Lymphocytes** | | | | | | |
| **IL-2** |  | | | | | |
| 1.1-9.8pg/ml | 54 | 119 |  |  | 12.307 | **0.002\*** |
| £¾9.8 pg/ml | 6 | 0 | 12.313 | **0.001\*** |  |  |
| £¼1.1 pg/ml | 6 | 11 | 0.119 | 0.786 |  |  |
| **IL-4** |  | | | | | |
| 0.1-3 pg/ml | 50 | 107 |  |  | 23.434 | **<0.001\*** |
| £¾3 pg/ml | 4 | 12 | 0.317 | 0.778 |  |  |
| £¼0.1 pg/ml | 12 | 0 | 22.293 | **<0.001\*** |  |  |
| **IL-6** |  | | | | | |
| 1.7-16.6 pg/ml | 52 | 110 |  |  | 24.007 | **<0.001\*** |
| £¾16.6 pg/ml | 2 | 9 | 0.929 | 0.506 |  |  |
| £¼1.7 pg/ml | 12 | 0 | 22.153 | **<0.001\*** |  |  |
| **IL-10** |  | | | | | |
| 2.6-4.9 pg/ml | 52 | 110 |  |  | 20.143 | **<0.001\*** |
| £¾4.9 pg/ml | 2 | 8 | 0.64 | 0.727 |  |  |
| £¼2.6v | 12 | 1 | 18.807 | **<0.001\*** |  |  |
| **TNF-¦Á** |  | | | | | |
| 0.1-5.2 pg/ml | 52 | 117 |  |  | 23.767 | **<0.001\*** |
| £¾5.2 pg/ml | 2 | 2 | 0.673 | 0.59 |  |  |
| £¼0.1 pg/ml | 12 | 0 | 23.495 | **<0.001\*** |  |  |
| **INF-¦Ã** |  | | | | | |
| 1.6-17.3 pg/ml | 54 | 41 |  |  | 11.435 | **0.003\*** |
| £¾17.3 pg/ml | 0 | 2 | 2.565 | 0.194 |  |  |
| £¼1.6 pg/ml | 12 | 0 | 8.396 | **0.003\*** |  |  |

*P*:
A comparison of different ranges of clinical indicators, and a comparison of
clinical indicators that are above or below the normal range with clinical
indicators that are within the normal range. *P*\*: Multiple list
comparisons between different ranges of clinical indicators.

  

**Table S4. The risk of clinical indicators in
stroke patients with comorbid hyperlipidemia.**

|  |  |  |  |  |  |  |
| --- | --- | --- | --- | --- | --- | --- |
| **Variable** | **Comorbidity** | | **¦Ö2** | ***P*** | **¦Ö2** | ***P*****\*** |
|  | **No** | **Yes** |  |  |  |  |
| **Age (years)** | ¡¡ | | | | | |
| ¡Ý60 | 42 | 41 | 0.308 | 0.579 | / | / |
| £¼60 | 24 | 19 |  |  |  |  |
| **Blood Lipid Profile** | | | | | | |
| **LDL** |  | | | | | |
| 1.89-4.21*mg/dl* | 52 | 43 |  |  | 6.182 | **0.045\*** |
| £¾4.21 *mg/dl* | 8 | 15 | 2.95 | 0.106 |  |  |
| £¼1.89 *mg/dl* | 6 | 1 | 2.551 | 0.137 |  |  |
| **HDL** |  | | | | | |
| 1.03-1.55 *mg/dl* | 32 | 43 |  |  | 8.806 | **0.012\*** |
| £¾1.55 *mg/dl* | 9 | 2 | 5.895 | **0.022\*** |  |  |
| £¼1.03 *mg/dl* | 25 | 14 | 4.716 | **0.048\*** |  |  |
| **Antiphospholipid Antibody** | | | | | | |
| **TG** |  | | | | | |
| ¡Ý1.70 *mg/dl* | 12 | 41 | 28.928 | **<0.001\*** | / | / |
| <1.70 *mg/dl* | 44 | 16 |  |  |  |  |
| **aPL-IgA** |  | | | | | |
| ¡Ý2.5 *mg/dl* | 6 | 5 | 0.017 | 1 | / | / |
| £¼2.5 *mg/dl* | 60 | 46 |  |  |  |  |
| **aPL-IgG** |  | | | | | |
| ¡Ý2.0 *mg/dl* | 20 | 16 | 0.015 | 1 | / | / |
| £¼2.0 *mg/dl* | 46 | 35 |  |  |  |  |
| **aPL-IgM** |  | | | | | |
| ¡Ý2.5 *mg/dl* | 26 | 34 | 8.565 | **0.003\*** | / | / |
| <2.5 *mg/dl* | 40 | 17 |  |  |  |  |
| **Anti-¦Â2GPI-IgA** |  | | | | | |
| ¡Ý2.0 *mg/dl* | 6 | 12 | 4.607 | **0.040\*** | / | / |
| £¼2.0 *mg/dl* | 60 | 39 |  |  |  |  |
| **Anti-¦Â2GPI-IgG** |  | | | | | |
| ¡Ý2.0 *mg/dl* | 19 | 19 | 0.94 | 0.426 | / | / |
| £¼2.0 *mg/dl* | 47 | 32 |  |  |  |  |
| **Anti-¦Â2GPI-IgM** |  | | | | | |
| ¡Ý2.0 *mg/dl* | 26 | 35 | 9.852 | **0.002\*** | / | / |
| £¼2.0 *mg/dl* | 40 | 16 |  |  |  |  |
| **Lymphocyte Subpopulation** | | | | | | |
| **CD3** |  | | | | | |
| 59.4-84.6% | 45 | 52 |  |  | 9.308 | **0.010\*** |
| £¾84.6% | 5 | 6 | 0.003 | 1 |  |  |
| £¼59.4% | 16 | 3 | 9.114 | **0.003\*** |  |  |
| **CD4** |  | | | | | |
| 28.5-60.5% | 52 | 56 |  |  | 4.558 | 0.102 |
| £¾60.5% | 6 | 3 | 1.14 | 0.322 |  |  |
| £¼28.5% | 8 | 2 | 3.715 | 0.095 |  |  |
| **CD8** |  | | | | | |
| 11.1-38.3% | 56 | 57 |  |  | 2.387 | 0.303 |
| £¾38.3% | 5 | 2 | 1.262 | 0.439 |  |  |
| £¼11.1% | 5 | 2 | 1.262 | 0.439 |  |  |
| **CD4/8** |  | | | | | |
| 1.1-2.5% | 36 | 36 |  |  | 0.346 | 0.841 |
| £¾2.5% | 23 | 20 | 0.131 | 0.847 |  |  |
| £¼1.1% | 7 | 5 | 0.286 | 0.757 |  |  |
| **CD19** |  | | | | | |
| 6.4-22.4% | 44 | 47 |  |  | 2.419 | 0.298 |
| £¾22.4% | 7 | 3 | 1.689 | 0.318 |  |  |
| £¼6.4% | 15 | 10 | 1.065 | 0.369 |  |  |
| **NK** |  | | | | | |
| 5.6-30.9% | 49 | 52 |  |  | 3.2 | 0.202 |
| £¾30.9% | 7 | 4 | 0.907 | 0.527 |  |  |
| £¼5.6% | 10 | 4 | 2.584 | 0.154 |  |  |
| **Th1/Th2 Lymphocytes** | | | | | | |
| **IL-2** |  | | | | | |
| 1.1-9.8pg/ml | 54 | 53 | 5.597 |  | 5.782 | 0.056 |
| £¾9.8 pg/ml | 6 | 0 | 0.333 | **0.029\*** |  |  |
| £¼1.1 pg/ml | 6 | 4 |  | 0.744 |  |  |
| **IL-4** |  | | | | | |
| 0.1-3 pg/ml | 50 | 49 |  |  | 10.718 | **0.005\*** |
| £¾3 pg/ml | 4 | 4 | 0.001 | 1 |  |  |
| £¼0.1 pg/ml | 12 | 0 | 10.633 | **0.001\*** |  |  |
| **IL-6** |  | | | | | |
| 1.7-16.6 pg/ml | 52 | 47 |  |  | 12.987 | **0.002\*** |
| £¾16.6 pg/ml | 2 | 6 | 2.243 | 0.161 |  |  |
| £¼1.7 pg/ml | 12 | 0 | 9.881 | **0.001\*** |  |  |
| **IL-10** |  | | | | | |
| 2.6-4.9 pg/ml | 52 | 51 |  |  | 7.826 | **0.020\*** |
| £¾4.9 pg/ml | 2 | 0 | 1.926 | 0.496 |  |  |
| £¼2.6 pg/ml | 12 | 2 | 6.173 | **0.020\*** |  |  |
| **TNF-¦Á** |  | | | | | |
| 0.1-5.2 pg/ml | 52 | 52 |  |  | 11.045 | **0.004\*** |
| £¾5.2 pg/ml | 2 | 1 | 0.324 | 1 |  |  |
| £¼0.1 pg/ml | 12 | 0 | 10.875 | **0.001\*** |  |  |
| **INF-¦Ã** |  | | | | | |
| 1.6-17.3 pg/ml | 54 | 53 |  |  | 10.717 | **0.001\*** |
| £¾17.3 pg/ml | 0 | 0 | / | / |  |  |
| £¼1.6 pg/ml | 12 | 0 | 10.717 | **0.001\*** |  |  |

*P*:
A comparison of different ranges of clinical indicators, and a comparison of
clinical indicators that are above or below the normal range with clinical
indicators that are within the normal range. *P*\*: Multiple list
comparisons between different ranges of clinical indicators.

  

**Table
S5. The risk of clinical
indicators in stroke patients with comorbid atrialfibrillation.**

|  |  |  |  |  |  |  |
| --- | --- | --- | --- | --- | --- | --- |
| **Variable** | **Comorbidity** | | **¦Ö2** | ***P*** | **¦Ö2** | ***P\**** |
|  | **No** | **Yes** |  |  |  |  |
| **Age (years)** | ¡¡ | | | | | |
| ¡Ý60 | 42 | 45 | 12.141 | **<0.001\*** | / | / |
| <60 | 24 | 4 |  |  |  |  |
| **Blood Lipid Profile** | | | | | | |
| **LDL** |  | | | | | |
| 1.89-4.21*mg/dl* | 52 | 30 |  |  | 8.772 | **0.012\*** |
| £¾4.21 *mg/dl* | 8 | 4 | 0.048 | 1 |  |  |
| £¼1.89 *mg/dl* | 6 | 15 | 8.25 | **0.006\*** |  |  |
| **HDL** |  | | | | | |
| 1.03-1.55 *mg/dl* | 32 | 17 |  |  | 9.325 | **0.009\*** |
| £¾1.55 *mg/dl* | 9 | 1 | 2.389 | 0.154 |  |  |
| £¼1.03 *mg/dl* | 25 | 31 | 4.496 | **0.034\*** |  |  |
| **TG** |  | | | | | |
| ¡Ý1.70 *mg/dl* | 12 | 5 | 2.426 | 0.184 | / | / |
| <1.70 *mg/dl* | 44 | 44 |  |  |  |  |
| **Antiphospholipid Antibody** | | | | | | |
| **aPL-IgA** |  | | | | | |
| ¡Ý2.5 *mg/dl* | 6 | 0 | 3.004 | 0.172 | / | / |
| £¼2.5 *mg/dl* | 60 | 31 |  |  |  |  |
| **aPL-IgG** |  | | | | | |
| ¡Ý2.0 *mg/dl* | 20 | 13 | 1.272 | 0.358 | / | / |
| £¼2.0 *mg/dl* | 46 | 18 |  |  |  |  |
| **aPL-IgM** |  | | | | | |
| ¡Ý2.5 *mg/dl* | 26 | 9 | 0.982 | 0.371 | / | / |
| £¼2.5 *mg/dl* | 40 | 22 |  |  |  |  |
| **Anti-¦Â2GPI-IgA** |  | | | | | |
| ¡Ý2.0 *mg/dl* | 6 | 6 | 2.05 | 0.19 | / | / |
| £¼2.0 *mg/dl* | 60 | 25 |  |  |  |  |
| **Anti-¦Â2GPI-IgG** |  | | | | | |
| ¡Ý2.0 *mg/dl* | 19 | 11 | 0.443 | 0.638 | / | / |
| £¼2.0 *mg/dl* | 47 | 20 |  |  |  |  |
| **Anti-¦Â2GPI-IgM** |  | | | | | |
| ¡Ý2.0 *mg/dl* | 26 | 13 | 0.057 | 0.828 | / | / |
| £¼2.0 *mg/dl* | 40 | 18 |  |  |  |  |
| **Lymphocyte Subpopulation** | | | | | | |
| **CD3** |  | | | | | |
| 59.4-84.6% | 45 | 34 |  |  | 2.063 | 0.356 |
| £¾84.6% | 5 | 1 | 1.601 | 0.393 |  |  |
| £¼59.4% | 16 | 15 | 0.258 | 0.672 |  |  |
| **CD4** |  | | | | | |
| 28.5-60.5% | 52 | 42 |  |  | 2.462 | 0.292 |
| £¾60.5% | 6 | 1 | 2.462 | 0.234 |  |  |
| £¼28.5% | 8 | 6 | 0.016 | 1 |  |  |
| **CD8** |  | | | | | |
| 11.1-38.3% | 56 | 45 |  |  | 1.893 | 0.388 |
| £¾38.3% | 5 | 1 | 1.797 | 0.234 |  |  |
| £¼11.1% | 5 | 3 | 0.15 | 1 |  |  |
| **CD4/8** |  | | | | | |
| 1.1-2.5% | 36 | 34 |  |  | 3.066 | 0.216 |
| £¾2.5% | 23 | 10 | 3.059 | 0.092 |  |  |
| £¼1.1% | 7 | 5 | 0.196 | 0.76 |  |  |
| **CD19** |  | | | | | |
| 6.4-22.4% | 44 | 33 |  |  | 0.314 | 0.855 |
| £¾22.4% | 7 | 6 | 0.049 | 1 |  |  |
| £¼6.4% | 15 | 9 | 0.216 | 0.813 |  |  |
| **NK** |  | | | | | |
| 5.6-30.9% | 49 | 34 |  |  | 3.046 | 0.218 |
| £¾30.9% | 7 | 10 | 1.827 | 0.192 |  |  |
| £¼5.6% | 10 | 4 | 0.772 | 0.556 |  |  |
| **Th1/Th2 Lymphocytes** | | | | | | |
| **IL-2** |  | | | | | |
| 1.1-9.8pg/ml | 54 | 36 |  |  | 3.855 | 0.146 |
| £¾9.8 pg/ml | 6 | 0 | 3.84 | 0.081 |  |  |
| £¼1.1 pg/ml | 6 | 4 | 0 | 1 |  |  |
| **IL-4** |  | | | | | |
| 0.1-3 pg/ml | 50 | 31 |  |  | 8.478 | **0.014\*** |
| £¾3 pg/ml | 4 | 5 | 1.008 | 0.475 |  |  |
| £¼0.1 pg/ml | 12 | 0 | 6.889 | **0.007\*** |  |  |
| **IL-6** |  | | | | | |
| 1.7-16.6 pg/ml | 52 | 32 |  |  | 9.42 | **0.009\*** |
| £¾16.6 pg/ml | 2 | 4 | 1.905 | 0.213 |  |  |
| £¼1.7 pg/ml | 12 | 0 | 6.857 | **0.007\*** |  |  |
| **IL-10** |  | | | | | |
| 2.6-4.9 pg/ml | 52 | 33 |  |  | 5.179 | 0.075 |
| £¾4.9 pg/ml | 2 | 2 | 0.2 | 0.644 |  |  |
| £¼2.6v | 12 | 1 | 4.823 | **0.031\*** |  |  |
| **TNF-¦Á** |  | | | | | |
| 0.1-5.2 pg/ml | 52 | 35 |  |  | 7.479 | **0.024\*** |
| £¾5.2 pg/ml | 2 | 1 | 0.057 | 1 |  |  |
| £¼0.1 pg/ml | 12 | 0 | 7.468 | **0.007\*** |  |  |
| **INF-¦Ã** |  | | | | | |
| 1.6-17.3 pg/ml | 54 | 35 | 1.517 | 0.4 | 9.012 | **0.011\*** |
| £¾17.3 pg/ml | 0 | 1 |  |  |  |  |
| £¼1.6 pg/ml | 12 | 0 | 7.222 | **0.007\*** |  |  |

*P*:
A comparison of different ranges of clinical indicators, and a comparison of
clinical indicators that are above or below the normal range with clinical
indicators that are within the normal range. *P*\*: Multiple list
comparisons between different ranges of clinical indicators.

  

**Table
S6. The risk of clinical
indicators in stroke patients with comorbid hyperhomocysteinaemia.**

|  |  |  |  |  |  |  |
| --- | --- | --- | --- | --- | --- | --- |
| **Variable** | **Comorbidity** | | **¦Ö2** | ***P*** | **¦Ö2** | ***P*****\*** |
|  | **No** | **Yes** |  |  |  |  |
| **Age (years)** | ¡¡ | | | | | |
| ¡Ý60 | 42 | 19 | 0.167 | 0.824 | / | / |
| £¼60 | 24 | 13 |  |  |  |  |
| **Blood Lipid Profile** | | | | | | |
| **LDL** |  | | | | | |
| 1.89-4.21*mg/dl* | 52 | 26 |  |  | 3.5 | 0.174 |
| £¾4.21 *mg/dl* | 8 | 1 | 1.862 | 0.263 |  |  |
| £¼1.89 *mg/dl* | 6 | 6 | 1.261 | 0.334 |  |  |
| **HDL** |  | | | | | |
| 1.03-1.55 *mg/dl* | 32 | 20 |  |  | 1.877 | 0.391 |
| £¾1.55 *mg/dl* | 9 | 2 | 1.643 | 0.302 |  |  |
| £¼1.03 *mg/dl* | 25 | 11 | 0.583 | 0.501 |  |  |
| **TG** |  | | | | | |
| ¡Ý1.70 *mg/dl* | 12 | 10 | 0.879 | 0.446 | / | / |
| <1.70 *mg/dl* | 44 | 23 |  |  |  |  |
| **Antiphospholipid Antibody** | | | | | | |
| **aPL-IgA** |  | | | | | |
| ¡Ý2.5 *mg/dl* | 6 | 0 | 2.719 | 0.174 | / | / |
| £¼2.5 *mg/dl* | 60 | 28 |  |  |  |  |
| **aPL-IgG** |  | | | | | |
| ¡Ý2.0 *mg/dl* | 20 | 8 | 0.028 | 1 | / | / |
| £¼2.0 *mg/dl* | 46 | 20 |  |  |  |  |
| **aPL-IgM** |  | | | | | |
| ¡Ý2.5 *mg/dl* | 26 | 12 | 0.098 | 0.82 | / | / |
| £¼2.5 *mg/dl* | 40 | 16 |  |  |  |  |
| **Anti-¦Â2GPI-IgA** |  | | | | | |
| ¡Ý2.0 *mg/dl* | 6 | 3 | 0.06 | 1 | / | / |
| £¼2.0 *mg/dl* | 60 | 25 |  |  |  |  |
| **Anti-¦Â2GPI-IgG** |  | | | | | |
| ¡Ý2.0 *mg/dl* | 19 | 8 | 0 | 1 | / | / |
| £¼2.0 *mg/dl* | 47 | 20 |  |  |  |  |
| **Anti-¦Â2GPI-IgM** |  | | | | | |
| ¡Ý2.0 *mg/dl* | 26 | 13 | 0.401 | 0.648 | / | / |
| £¼2.0 *mg/dl* | 40 | 15 |  |  |  |  |
| **Lymphocyte Subpopulation** | | | | | | |
| **CD3** |  | | | | | |
| 59.4-84.6% | 45 | 29 |  |  | 3.788 | 0.15 |
| £¾84.6% | 5 | 2 | 0.305 | 0.702 |  |  |
| £¼59.4% | 16 | 3 | 3.668 | 0.063 |  |  |
| **CD4** |  | | | | | |
| 28.5-60.5% | 52 | 31 |  |  | 2.501 | 0.286 |
| £¾60.5% | 6 | 1 | 1.499 | 0.414 |  |  |
| £¼28.5% | 8 | 2 | 1.173 | 0.486 |  |  |
| **CD8** |  | | | | | |
| 11.1-38.3% | 56 | 29 |  |  | 0.136 | 0.934 |
| £¾38.3% | 5 | 2 | 0.089 | 1 |  |  |
| £¼11.1% | 5 | 3 | 0.037 | 1 |  |  |
| **CD4/8** |  | | | | | |
| 1.1-2.5% | 36 | 19 |  |  | 0.523 | 0.77 |
| £¾2.5% | 23 | 10 | 0.168 | 0.816 |  |  |
| £¼1.1% | 7 | 5 | 0.217 | 0.743 |  |  |
| **CD19** |  | | | | | |
| 6.4-22.4% | 44 | 24 |  |  | 1.83 | 0.401 |
| £¾22.4% | 7 | 1 | 1.685 | 0.259 |  |  |
| £¼6.4% | 15 | 9 | 0.038 | 1 |  |  |
| **NK** |  | | | | | |
| 5.6-30.9% | 49 | 29 |  |  | 1.599 | 0.45 |
| £¾30.9% | 7 | 2 | 0.787 | 0.481 |  |  |
| £¼5.6% | 10 | 3 | 0.972 | 0.531 |  |  |
| **Th1/Th2 Lymphocytes** | | | | | | |
| **IL-2** |  | | | | | |
| 1.1-9.8pg/ml | 54 | 27 |  |  | 2.909 | 0.234 |
| £¾9.8 pg/ml | 6 | 0 | 2.9 | 0.171 |  |  |
| £¼1.1 pg/ml | 6 | 3 | 0 | 1 |  |  |
| **IL-4** |  | | | | | |
| 0.1-3 pg/ml | 50 | 27 |  |  | 6.043 | **0.049\*** |
| £¾3 pg/ml | 4 | 2 | 0.007 | 1 |  |  |
| £¼0.1 pg/ml | 12 | 0 | 6.04 | **0.015\*** |  |  |
| **IL-6** |  | | | | | |
| 1.7-16.6 pg/ml | 52 | 28 |  |  | 6.039 | **0.049\*** |
| £¾16.6 pg/ml | 2 | 1 | 0.004 | 1 |  |  |
| £¼1.7 pg/ml | 12 | 0 | 6.038 | **0.015\*** |  |  |
| **IL-10** |  | | | | | |
| 2.6-4.9 pg/ml | 52 | 26 |  |  | 2.042 | 0.36 |
| £¾4.9 pg/ml | 2 | 1 | 0 | 1 |  |  |
| £¼2.6 pg/ml | 12 | 2 | 2.034 | 0.213 |  |  |
| **TNF-¦Á** |  | | | | | |
| 0.1-5.2 pg/ml | 52 | 29 |  |  | 7.215 | **0.027\*** |
| £¾5.2 pg/ml | 2 | 0 | 1.101 | 0.54 |  |  |
| £¼0.1 pg/ml | 12 | 0 | 6.243 | **0.016\*** |  |  |
| **INF-¦Ã** |  | | | | | |
| 1.6-17.3 pg/ml | 54 | 27 | / | / | 2.042 | 0.214 |
| £¾17.3 pg/ml | 0 | 0 |  |  |  |  |
| £¼1.6 pg/ml | 12 | 2 | 2.042 | 0.214 |  |  |

*P*:
A comparison of different ranges of clinical indicators, and a comparison of
clinical indicators that are above or below the normal range with clinical
indicators that are within the normal range. *P*\*: Multiple list
comparisons between different ranges of clinical indicators.

  

**Table
S7. The risk of clinical
indicators in stroke patients with comorbid multiple disease.**

|  |  |  |  |  |  |  |
| --- | --- | --- | --- | --- | --- | --- |
| **Variable** | **Comorbidity** | | **¦Ö2** | ***P*** | **¦Ö2** | ***P*****\*** |
|  | **No** | **Yes** |  |  |  |  |
| **Age (years)** | ¡¡ | | | | | |
| ¡Ý60 | 42 | 267 | 1.878 | 0.171 | / | / |
| £¼60 | 24 | 104 |  |  |  |  |
| **Blood Lipid Profile** | | | | | | |
| **LDL** |  | | | | | |
| 1.89-4.21*mg/dl* | 52 | 288 |  |  | 3.283 | 0.194 |
| £¾4.21 *mg/dl* | 8 | 22 | 2.624 | 0.12 |  |  |
| £¼1.89 *mg/dl* | 6 | 44 | 0.374 | 0.541 |  |  |
| **HDL** |  | | | | | |
| 1.03-1.55 *mg/dl* | 32 | 198 |  |  | 7.604 | **0.022\*** |
| £¾1.55 *mg/dl* | 9 | 17 | 7.443 | 0.02 |  |  |
| £¼1.03 *mg/dl* | 25 | 139 | 0.137 | 0.936 |  |  |
| **TG** |  | | | | | |
| ¡Ý1.70 *mg/dl* | 12 | 94 | 0.663 | 0.416 | / | / |
| <1.70 *mg/dl* | 44 | 260 |  |  |  |  |
| **Antiphospholipid Antibody** | | | | | | |
| **aPL-IgA** |  | | | | | |
| ¡Ý2.5 *mg/dl* | 6 | 17 | 0.813 | 0.407 | / | / |
| £¼2.5 *mg/dl* | 60 | 265 |  |  |  |  |
| **aPL-IgG** |  | | | | | |
| ¡Ý2.0 *mg/dl* | 20 | 82 | 0.032 | 0.857 | / | / |
| £¼2.0 *mg/dl* | 46 | 199 |  |  |  |  |
| **aPL-IgM** |  | | | | | |
| ¡Ý2.5 *mg/dl* | 26 | 129 | 0.917 | 0.338 | / | / |
| £¼2.5 *mg/dl* | 40 | 152 |  |  |  |  |
| **Anti-¦Â2GPI-IgA** |  | | | | | |
| ¡Ý2.0 *mg/dl* | 6 | 41 | 1.381 | 0.24 | / | / |
| £¼2.0 *mg/dl* | 60 | 240 |  |  |  |  |
| **Anti-¦Â2GPI-IgG** |  | | | | | |
| ¡Ý2.0 *mg/dl* | 19 | 85 | 0.054 | 0.816 | / | / |
| £¼2.0 *mg/dl* | 47 | 196 |  |  |  |  |
| **Anti-¦Â2GPI-IgM** |  | | | | | |
| ¡Ý2.0 *mg/dl* | 26 | 123 | 0.418 | 0.518 | / | / |
| £¼2.0 *mg/dl* | 40 | 158 |  |  |  |  |
| **Lymphocyte Subpopulation** | | | | | | |
| **CD3** |  | | | | | |
| 59.4-84.6% | 45 | 283 |  |  | 2.147 | 0.342 |
| £¾84.6% | 5 | 23 | 0.366 | 0.569 |  |  |
| £¼59.4% | 16 | 64 | 1.995 | 0.158 |  |  |
| **CD4** |  | | | | | |
| 28.5-60.5% | 52 | 317 |  |  | 4.144 | 0.126 |
| £¾60.5% | 6 | 19 | 1.831 | 0.236 |  |  |
| £¼28.5% | 8 | 24 | 2.754 | 0.118 |  |  |
| **CD8** |  | | | | | |
| 11.1-38.3% | 56 | 328 |  |  | 2.461 | 0.292 |
| £¾38.3% | 5 | 16 | 1.325 | 0.224 |  |  |
| £¼11.1% | 5 | 16 | 1.325 | 0.224 |  |  |
| **CD4/8** |  | | | | | |
| 1.1-2.5% | 36 | 202 |  |  | 0.091 | 0.956 |
| £¾2.5% | 23 | 119 | 0.078 | 0.78 |  |  |
| £¼1.1% | 7 | 40 | 0.002 | 0.968 |  |  |
| **CD19** |  | | | | | |
| 6.4-22.4% | 44 | 279 |  |  | 4.489 | 0.106 |
| £¾22.4% | 7 | 26 | 1.405 | 0.292 |  |  |
| £¼6.4% | 15 | 50 | 3.751 | 0.053 |  |  |
| **NK** |  | | | | | |
| 5.6-30.9% | 49 | 287 |  |  | 3.558 | 0.169 |
| £¾30.9% | 7 | 39 | 0.013 | 0.909 |  |  |
| £¼5.6% | 10 | 28 | 3.537 | 0.096 |  |  |
| **Th1/Th2 Lymphocytes** | | | | | | |
| **IL-2** |  | | | | | |
| 1.1-9.8pg/ml | 54 | 272 |  |  | 27.826 | **<0.001\*** |
| £¾9.8 pg/ml | 6 | 0 | 27.701 | **<0.001\*** |  |  |
| £¼1.1 pg/ml | 6 | 29 | 0.008 | 1 |  |  |
| **IL-4** |  | | | | | |
| 0.1-3 pg/ml | 50 | 244 |  |  | 51.648 | **<0.001\*** |
| £¾3 pg/ml | 4 | 28 | 0.424 | 0.624 |  |  |
| £¼0.1 pg/ml | 12 | 0 | 49.153 | **<0.001\*** |  |  |
| **IL-6** |  | | | | | |
| 1.7-16.6 pg/ml | 52 | 257 |  |  | 51.538 | **<0.001\*** |
| £¾16.6 pg/ml | 2 | 15 | 0.299 | 0.748 |  |  |
| £¼1.7 pg/ml | 12 | 0 | 50.059 | **<0.001\*** |  |  |
| **IL-10** |  | | | | | |
| 2.6-4.9 pg/ml | 52 | 254 |  |  | 33.086 | **<0.001\*** |
| £¾4.9 pg/ml | 2 | 14 | 0.22 | 1 |  |  |
| £¼2.6v | 12 | 4 | 32.126 | **<0.001\*** |  |  |
| **TNF-¦Á** |  | | | | | |
| 0.1-5.2 pg/ml | 52 | 267 |  |  | 51.931 | **<0.001\*** |
| £¾5.2 pg/ml | 2 | 5 | 0.746 | 0.327 |  |  |
| £¼0.1 pg/ml | 12 | 0 | 51.946 | **<0.001\*** |  |  |
| **INF-**¦Ã |  | | | | | |
| 1.6-17.3 pg/ml | 54 | 270 |  |  | 51.626 | **<0.001\*** |
| £¾17.3 pg/ml | 0 | 2 | 0.4 | 1 |  |  |
| £¼1.6 pg/ml | 12 | 0 | 50.909 | **<0.001\*** |  |  |

*P*:
A comparison of different ranges of clinical indicators, and a comparison of
clinical indicators that are above or below the normal range with clinical
indicators that are within the normal range. *P*\*: Multiple list
comparisons between different ranges of clinical indicators.

 

  

 

**Table
S8. The risk of clinical indicators in stroke patients with lacunar infarction.**

|  |  |  |  |  |  |  |
| --- | --- | --- | --- | --- | --- | --- |
| **Variable** | **Stroke Subtypes** | | ***¦Ö²*** | ***P*****1** | ***¦Ö²*** | ***P*****2** |
|  | **No** | **Yes** |  |  |  |  |
| **Age (years)** |  |  |  | ¡¡ | ¡¡ | ¡¡ |
| ¡Ý60 | 236 | 55 | 3.552 | 0.059 | / | / |
| £¼60 | 85 | 32 |  |  |  |  |
| **LDL** |  |  |  |  |  |  |
| 1.89-4.21*mg/dl* | 250 | 72 |  |  | 0.734 | 0.693 |
| £¾4.21 *mg/dl* | 20 | 7 | 0.181 | 0.637 |  |  |
| £¼1.89 *mg/dl* | 33 | 7 | 0.493 | 0.483 |  |  |
| **HDL** |  |  |  |  |  |  |
| 1.03-1.55 *mg/dl* | 168 | 52 |  |  | 1.630 | 0.443 |
| £¾1.55 *mg/dl* | 21 | 3 | 1.537 | 0.305 |  |  |
| £¼1.03 *mg/dl* | 114 | 31 | 0.253 | 0.615 |  |  |
| **TG** |  |  |  |  |  |  |
| ¡Ý1.70 *mg/dl* | 73 | 26 | 1.331 | 0.249 | / | / |
| <1.70 *mg/dl* | 230 | 60 |  |  |  |  |
| **Apl-IgA** |  |  |  |  |  |  |
| ¡Ý2.5 *mg/dl* | 20 | 4 | 0.615 | 0.615 | / | / |
| £¼2.5 *mg/dl* | 219 | 68 |  |  |  |  |
| **Apl-IgG** |  |  |  |  |  |  |
| ¡Ý2.0 *mg/dl* | 70 | 25 | 0.733 | 0.392 | / | / |
| £¼2.0 *mg/dl* | 168 | 47 |  |  |  |  |
| **Apl-IgM** |  |  |  |  |  |  |
| ¡Ý2.5 *mg/dl* | 108 | 38 | 1.215 | 0.270 | / | / |
| £¼2.5 *mg/dl* | 130 | 34 |  |  |  |  |
| **Anti-¦Â2GPI-IgA** |  |  |  |  |  |  |
| ¡Ý2.0 *mg/dl* | 35 | 9 | 0.260 | 0.610 | / | / |
| £¼2.0 *mg/dl* | 203 | 64 |  |  |  |  |
| **Anti-¦Â2GPI-IgG** |  |  |  |  |  |  |
| ¡Ý2.0 *mg/dl* | 73 | 24 | 0.182 | 0.670 | / | / |
| £¼2.0 *mg/dl* | 165 | 48 |  |  |  |  |
| **Anti-¦Â2GPI-IgM** |  |  |  |  |  |  |
| ¡Ý2.0 *mg/dl* | 102 | 37 | 1.627 | 0.202 | / | / |
| £¼2.0 *mg/dl* | 136 | 35 |  |  |  |  |
| **CD3** |  |  |  |  |  |  |
| 59.4-84.6% | 241 | 69 |  |  | 9.028 | **0.011\*** |
| £¾84.6% | 15 | 6 | 0.447 | 0.589 |  |  |
| £¼59.4% | 58 | 4 | 8.184 | **0.004\*** |  |  |
| **CD4** |  |  |  |  |  |  |
| 28.5-60.5% | 266 | 80 |  |  | 7.272 | **0.026\*** |
| £¾60.5% | 19 | 3 | 1.065 | 0.432 |  |  |
| £¼28.5% | 29 | 1 | 6.396 | **0.009\*** |  |  |
| **CD8** |  |  |  |  |  |  |
| 11.1-38.3% | 277 | 81 |  |  | 5.200 | 0.074 |
| £¾38.3% | 16 | 2 | 1.321 | 0.383 |  |  |
| £¼11.1% | 21 | 1 | 4.004 | 0.058 |  |  |
| **CD4/8** |  |  |  |  |  |  |
| 1.1-2.5% | 166 | 54 |  |  | 3.655 | 0.161 |
| £¾2.5% | 114 | 22 | 3.506 | 0.061 |  |  |
| £¼1.1% | 34 | 8 | 0.590 | 0.442 |  |  |
| **CD19** |  |  |  |  |  |  |
| 6.4-22.4% | 232 | 72 |  |  | 5.205 | 0.074 |
| £¾22.4% | 29 | 2 | 4.854 | **0.024\*** |  |  |
| £¼6.4% | 43 | 10 | 0.592 | 0.442 |  |  |
| **NK** |  |  |  |  |  |  |
| 5.6-30.9% | 244 | 72 |  |  | 1.830 | 0.401 |
| £¾30.9% | 38 | 7 | 1.204 | 0.272 |  |  |
| £¼5.6% | 22 | 4 | 0.761 | 0.470 |  |  |
| **IL-2** |  |  |  |  |  |  |
| 1.1-9.8pg/ml | 231 | 67 |  |  | / | **/** |
| £¾9.8 pg/ml | 0 | 0 | / | **/** |  |  |
| £¼1.1 pg/ml | 0 | 0 | / | / |  |  |
| **IL-4** |  |  |  |  |  |  |
| 0.1-3 pg/ml | 206 | 62 |  |  | 0.648 | 0.497 |
| £¾3 pg/ml | 25 | 5 | 0.648 | 0.497 |  |  |
| £¼0.1 pg/ml | 0 | 0 | / | **/** |  |  |
| **IL-6** |  |  |  |  |  |  |
| 1.7-16.6 pg/ml | 218 | 65 |  |  | 0.759 | 0.534 |
| £¾16.6 pg/ml | 13 | 2 | 0.759 | 0.534 |  |  |
| £¼1.7 pg/ml | 0 | 0 | / | **/** |  |  |
| **IL-10** |  |  |  |  |  |  |
| 2.6-4.9 pg/ml | 216 | 64 |  |  | 0.946 | 0.623 |
| £¾4.9 pg/ml | 13 | 2 | 0.744 | 0.534 |  |  |
| £¼2.6 pg/ml | 2 | 1 | 0.184 | 0.544 |  |  |
| **TNF-¦Á** |  |  |  |  |  |  |
| 0.1-5.2 pg/ml | 227 | 64 |  |  | 1.707 | 0.191 |
| £¾5.2 pg/ml | 4 | 3 | 1.707 | 0.191 |  |  |
| £¼0.1 pg/ml | 0 | 0 | / | **/** |  |  |
| **INF-¦Ã** |  |  |  |  |  |  |
| 1.6-17.3 pg/ml | 230 | 67 |  |  | 13.471 | **0.001\*** |
| £¾17.3 pg/ml | 1 | 0 | 0.291 | 1.000 |  |  |
| £¼1.6 pg/ml | 0 | 4 | 13.132 | **0.003\*** |  |  |

  

*P*:
A comparison of different ranges of clinical indicators, and a comparison of
clinical indicators that are above or below the normal range with clinical
indicators that are within the normal range. *P*\*: Multiple list
comparisons between different ranges of clinical indicators.

  

**Table
S9. The risk of clinical indicators in stroke patients with cardiogenic
embolism.**

|  |  |  |  |  |  |  |
| --- | --- | --- | --- | --- | --- | --- |
| **Variable** | **Stroke Subtypes** | | ***¦Ö²*** | ***P*****1** | ***¦Ö²*** | ***P*****2** |
|  | **No** | **Yes** |  |  |  |  |
| **Age (years)** |  |  |  | ¡¡ | ¡¡ | ¡¡ |
| ¡Ý60 | 236 | 7 | 2.502 | 0.252 | / | / |
| £¼60 | 85 | 0 |  |  |  |  |
| **LDL** |  |  |  |  |  |  |
| 1.89-4.21*mg/dl* | 250 | 3 |  |  | 7.935 | **0.019\*** |
| £¾4.21 *mg/dl* | 20 | 1 | 1.724 | 0.274 |  |  |
| £¼1.89 *mg/dl* | 33 | 3 | 7.920 | **0.027\*** |  |  |
| **HDL** |  |  |  |  |  |  |
| 1.03-1.55 *mg/dl* | 168 | 3 |  |  | 1.367 | 0.505 |
| £¾1.55 *mg/dl* | 21 | 0 | 0.374 | 1.000 |  |  |
| £¼1.03 *mg/dl* | 114 | 4 | 0.790 | 0.617 |  |  |
| **TG** |  |  |  |  |  |  |
| ¡Ý1.70 *mg/dl* | 73 | 2 | 0.075 | 1.000 | / | / |
| <1.70 *mg/dl* | 230 | 5 |  |  |  |  |
| **aPL-IgA** |  |  |  |  |  |  |
| ¡Ý2.5 *mg/dl* | 20 | 0 | 0.274 | 1.000 | / | / |
| £¼2.5 *mg/dl* | 219 | 3 |  |  |  |  |
| **aPL-IgG** |  |  |  |  |  |  |
| ¡Ý2.0 *mg/dl* | 70 | 2 | 1.963 | 0.443 | / | / |
| £¼2.0 *mg/dl* | 168 | 1 |  |  |  |  |
| **aPL-IgM** |  |  |  |  |  |  |
| ¡Ý2.5 *mg/dl* | 108 | 2 | 0.541 | 0.879 | / | / |
| £¼2.5 *mg/dl* | 130 | 1 |  |  |  |  |
| **Anti-¦Â2GPI-IgA** |  |  |  |  |  |  |
| ¡Ý2.0 *mg/dl* | 35 | 0 | 0.516 | 1.000 | / | / |
| £¼2.0 *mg/dl* | 203 | 3 |  |  |  |  |
| **Anti-¦Â2GPI-IgG** |  |  |  |  |  |  |
| ¡Ý2.0 *mg/dl* | 73 | 2 | 1.791 | 0.477 | / | / |
| £¼2.0 *mg/dl* | 165 | 1 |  |  |  |  |
| **Anti-¦Â2GPI-IgM** |  |  |  |  |  |  |
| ¡Ý2.0 *mg/dl* | 102 | 2 | 0.685 | 0.810 | / | / |
| £¼2.0 *mg/dl* | 136 | 1 |  |  |  |  |
| **CD3** |  |  |  |  |  |  |
| 59.4-84.6% | 241 | 4 |  |  | 1.961 | 0.375 |
| £¾84.6% | 15 | 1 | 1.704 | 0.273 |  |  |
| £¼59.4% | 58 | 2 | 0.723 | 0.740 |  |  |
| **CD4** |  |  |  |  |  |  |
| 28.5-60.5% | 266 | 5 |  |  | 1.075 | 0.584 |
| £¾60.5% | 19 | 1 | 0.918 | 0.350 |  |  |
| £¼28.5% | 29 | 1 | 0.306 | 0.470 |  |  |
| **CD8** |  |  |  |  |  |  |
| 11.1-38.3% | 277 | 5 |  |  | 1.888 | 0.389 |
| £¾38.3% | 16 | 1 | 1.377 | 0.298 |  |  |
| £¼11.1% | 21 | 1 | 0.811 | 0.365 |  |  |
| **CD4/8** |  |  |  |  |  |  |
| 1.1-2.5% | 166 | 3 |  |  | 0.286 | 0.867 |
| £¾2.5% | 114 | 3 | 0.210 | 0.970 |  |  |
| £¼1.1% | 34 | 1 | 0.177 | 0.532 |  |  |
| **CD19** |  |  |  |  |  |  |
| 6.4-22.4% | 232 | 5 |  |  | 1.673 | 0.433 |
| £¾22.4% | 29 | 0 | 0.624 | 1.000 |  |  |
| £¼6.4% | 43 | 2 | 0.852 | 0.689 |  |  |
| **NK** |  |  |  |  |  |  |
| 5.6-30.9% | 244 | 4 |  |  | 2.292 | 0.318 |
| £¾30.9% | 38 | 2 | 1.937 | 0.426 |  |  |
| £¼5.6% | 22 | 1 | 0.869 | 0.360 |  |  |
| **IL-2** |  |  |  |  |  |  |
| 1.1-9.8pg/ml | 231 | 6 |  |  | / | / |
| £¾9.8 pg/ml | 0 | 0 | / | / |  |  |
| £¼1.1 pg/ml | 0 | 0 | / | / |  |  |
| **IL-4** |  |  |  |  |  |  |
| 0.1-3 pg/ml | 206 | 5 |  |  | 0.205 | 0.506 |
| £¾3 pg/ml | 25 | 1 | 0.205 | 0.506 |  |  |
| £¼0.1 pg/ml | 0 | 0 | / | / |  |  |
| **IL-6** |  |  |  |  |  |  |
| 1.7-16.6 pg/ml | 218 | 6 |  |  | 0.357 | 1.000 |
| £¾16.6 pg/ml | 13 | 0 | 0.357 | 1.000 |  |  |
| £¼1.7 pg/ml | 0 | 0 | / | / |  |  |
| **IL-10** |  |  |  |  |  |  |
| 2.6-4.9 pg/ml | 216 | 5 |  |  | 11.937 | **0.003\*** |
| £¾4.9 pg/ml | 13 | 0 | 0.301 | 1.000 |  |  |
| £¼2.6 pg/ml | 2 | 1 | 10.961 | 0.079 |  |  |
| **TNF-¦Á** |  |  |  |  |  |  |
| 0.1-5.2 pg/ml | 227 | 6 |  |  | 0.106 | 1.000 |
| £¾5.2 pg/ml | 4 | 0 | 0.106 | 1.000 |  |  |
| £¼0.1 pg/ml | 0 | 0 | / | / |  |  |
| **INF-¦Ã** |  |  |  |  |  |  |
| 1.6-17.3 pg/ml | 230 | 5 |  |  | 18.418 | **0.050\*** |
| £¾17.3 pg/ml | 1 | 1 | 18.418 | **0.050\*** |  |  |
| £¼1.6 pg/ml | 0 | 0 | / | / |  |  |

*P*:
A comparison of different ranges of clinical indicators, and a comparison of
clinical indicators that are above or below the normal range with clinical
indicators that are within the normal range. *P*\*: Multiple list
comparisons between different ranges of clinical indicators.

  

**Table
S10. The risk of clinical indicators in stroke patients with aorta
atherosclerosis.**

|  |  |  |  |  |  |  |
| --- | --- | --- | --- | --- | --- | --- |
| **Variable** | **Stroke Subtypes** | | ***¦Ö²*** | ***P*****1** | ***¦Ö²*** | ***P*****2** |
|  | **No** | **Yes** |  |  |  |  |
| **Age (years)** |  |  |  | ¡¡ | ¡¡ | ¡¡ |
| ¡Ý60 | 236 | 15 | 1.368 | 0.242 | / | / |
| £¼60 | 85 | 9 |  |  |  |  |
| **LDL** |  |  |  |  |  |  |
| 1.89-4.21*mg/dl* | 250 | 18 |  |  | 1.011 | 0.603 |
| £¾4.21 *mg/dl* | 20 | 1 | 0.121 | 1.000 |  |  |
| £¼1.89 *mg/dl* | 33 | 4 | 0.814 | 0.322 |  |  |
| **HDL** |  |  |  |  |  |  |
| 1.03-1.55 *mg/dl* | 168 | 13 |  |  | 2.062 | 0.357 |
| £¾1.55 *mg/dl* | 21 | 0 | 1.612 | 0.369 |  |  |
| £¼1.03 *mg/dl* | 114 | 11 | 0.268 | 0.605 |  |  |
| **TG** |  |  |  |  |  |  |
| ¡Ý1.70 *mg/dl* | 73 | 6 | 0.010 | 0.920 | / | / |
| <1.70 *mg/dl* | 230 | 18 |  |  |  |  |
| **Apl-IgA** |  |  |  |  |  |  |
| ¡Ý2.5 *mg/dl* | 20 | 0 | 1.904 | 0.384 | / | / |
| £¼2.5 *mg/dl* | 219 | 21 |  |  |  |  |
| **Apl-IgG** |  |  |  |  |  |  |
| ¡Ý2.0 *mg/dl* | 70 | 5 | 0.294 | 0.587 | / | / |
| £¼2.0 *mg/dl* | 168 | 16 |  |  |  |  |
| **Apl-IgM** |  |  |  |  |  |  |
| ¡Ý2.5 *mg/dl* | 108 | 6 | 2.212 | 0.137 | / | / |
| £¼2.5 *mg/dl* | 130 | 15 |  |  |  |  |
| **Anti-¦Â2GPI-IgA** |  |  |  |  |  |  |
| ¡Ý2.0 *mg/dl* | 35 | 4 | 0.284 | 0.534 | / | / |
| £¼2.0 *mg/dl* | 203 | 17 |  |  |  |  |
| **Anti-¦Â2GPI-IgG** |  |  |  |  |  |  |
| ¡Ý2.0 *mg/dl* | 73 | 6 | 0.040 | 0.841 | / | / |
| £¼2.0 *mg/dl* | 165 | 15 |  |  |  |  |
| **Anti-¦Â2GPI-IgM** |  |  |  |  |  |  |
| ¡Ý2.0 *mg/dl* | 102 | 7 | 0.718 | 0.397 | / | / |
| £¼2.0 *mg/dl* | 136 | 14 |  |  |  |  |
| **CD3** |  |  |  |  |  |  |
| 59.4-84.6% | 241 | 20 |  |  | 5.236 | 0.073 |
| £¾84.6% | 15 | 3 | 1.805 | 0.176 |  |  |
| £¼59.4% | 58 | 1 | 2.795 | 0.167 |  |  |
| **CD4** |  |  |  |  |  |  |
| 28.5-60.5% | 266 | 22 |  |  | 0.906 | 0.636 |
| £¾60.5% | 19 | 1 | 0.188 | 1.000 |  |  |
| £¼28.5% | 29 | 1 | 0.751 | 0.709 |  |  |
| **CD8** |  |  |  |  |  |  |
| 11.1-38.3% | 277 | 23 |  |  | 1.789 | 0.409 |
| £¾38.3% | 16 | 1 | 0.073 | 1.000 |  |  |
| £¼11.1% | 21 | 0 | 1.734 | 0.381 |  |  |
| **CD4/8** |  |  |  |  |  |  |
| 1.1-2.5% | 166 | 15 |  |  | 1.396 | 0.497 |
| £¾2.5% | 114 | 8 | 0.311 | 0.577 |  |  |
| £¼1.1% | 34 | 1 | 1.261 | 0.479 |  |  |
| **CD19** |  |  |  |  |  |  |
| 6.4-22.4% | 232 | 17 |  |  | 0.303 | 0.859 |
| £¾22.4% | 29 | 3 | 0.278 | 0.485 |  |  |
| £¼6.4% | 43 | 3 | 0.006 | 1.000 |  |  |
| **NK** |  |  |  |  |  |  |
| 5.6-30.9% | 244 | 19 |  |  | 5.663 | 0.059 |
| £¾30.9% | 38 | 0 | 2.930 | 0.146 |  |  |
| £¼5.6% | 22 | 4 | 2.151 | 0.139 |  |  |
| **IL-2** |  |  |  |  |  |  |
| 1.1-9.8pg/ml | 231 | 22 |  |  | / | **/** |
| £¾9.8 pg/ml | 0 | 0 | / | **/** |  |  |
| £¼1.1 pg/ml | 0 | 0 | / | / |  |  |
| **IL-4** |  |  |  |  |  |  |
| 0.1-3 pg/ml | 206 | 20 |  |  | 0.063 | 1.000 |
| £¾3 pg/ml | 25 | 2 | 0.063 | 1.000 |  |  |
| £¼0.1 pg/ml | 0 | 0 | / | / |  |  |
| **IL-6** |  |  |  |  |  |  |
| 1.7-16.6 pg/ml | 218 | 21 |  |  | 0.045 | 1.000 |
| £¾16.6 pg/ml | 13 | 1 | 0.045 | 1.000 |  |  |
| £¼1.7 pg/ml | 0 | 0 | / | / |  |  |
| **IL-10** |  |  |  |  |  |  |
| 2.6-4.9 pg/ml | 216 | 21 |  |  | 0.241 | 0.886 |
| £¾4.9 pg/ml | 13 | 1 | 0.049 | 1.000 |  |  |
| £¼2.6 pg/ml | 2 | 0 | 0.194 | 1.000 |  |  |
| **TNF-¦Á** |  |  |  |  |  |  |
| 0.1-5.2 pg/ml | 227 | 22 |  |  | 0.387 | 1.000 |
| £¾5.2 pg/ml | 4 | 0 | 0.387 | 1.000 |  |  |
| £¼0.1 pg/ml | 0 | 0 | / | **/** |  |  |
| **INF-¦Ã** |  |  |  |  |  |  |
| 1.6-17.3 pg/ml | 230 | 22 |  |  | 0.096 | 1.000 |
| £¾17.3 pg/ml | 1 | 0 | 0.096 | 1.000 |  |  |
| £¼1.6 pg/ml | 0 | 0 | / | **/** |  |  |

  

*P*:
A comparison of different ranges of clinical indicators, and a comparison of
clinical indicators that are above or below the normal range with clinical
indicators that are within the normal range. *P*\*: Multiple list
comparisons between different ranges of clinical indicators.
